# Multiple Neural Mechanisms of Decision Making and Their Competition under Changing Risk Pressure

**DOI:** 10.1016/j.neuron.2014.01.033

**Published:** 2014-03-05

**Authors:** Nils Kolling, Marco Wittmann, Matthew F.S. Rushworth

**Affiliations:** 1 Department of Experimental Psychology, University of Oxford, Oxford OX1 3UD, UK; 2 Centre for Functional MRI of the Brain, University of Oxford, Oxford OX3 9DU, UK

## Abstract

Sometimes when a choice is made, the outcome is not guaranteed and there is only a probability of its occurrence. Each individual’s attitude to probability, sometimes called risk proneness or aversion, has been assumed to be static. Behavioral ecological studies, however, suggest such attitudes are dynamically modulated by the context an organism finds itself in; in some cases, it may be optimal to pursue actions with a low probability of success but which are associated with potentially large gains. We show that human subjects rapidly adapt their use of probability as a function of current resources, goals, and opportunities for further foraging. We demonstrate that dorsal anterior cingulate cortex (dACC) carries signals indexing the pressure to pursue unlikely choices and signals related to the taking of such choices. We show that dACC exerts this control over behavior when it, rather than ventromedial prefrontal cortex, interacts with posterior cingulate cortex.

## Highlights

• Against common belief, risk preference is not fixed but is dynamically updated
• Environments exert varying “risk pressure” tracked by ACC
• Risk-related values and behavior are then regulated by ACC
• Two mechanisms regulate competition for control of behavior between ACC and vmPFC

## Teaser

The propensity to take risks has been considered a fixed personality trait. By contrast, Kolling et al. show that people modulate their degree of risk taking from moment to moment and that this ability is related to a suite of signals in the anterior cingulate cortex.

## Introduction

An understanding of risk and opportunity is essential for success and survival, and there has been interest in the neural representation of risk, probability, and value (Platt and Huettel, 2008). We know that individuals differ in attitudes to risk and probability. For example, people prepared to pursue a course of action that might lead to great potential gain (a large reward magnitude) even if there is a low probability of obtaining the outcome, are said to be risk prone, while others are called risk averse. Such variation in attitudes is linked to individual differences in brain activity (Tobler et al., 2007, 2009). It is recognized that such attitudes differ depending on the type of prospect contemplated—for example, whether it is a potential gain or a loss (Kahneman and Tversky, 2000)—but within a given frame, there has been less investigation of how the use of probability to guide behavior changes with circumstances. Despite the existence of individual differences in risk attitudes, it is possible that how each individual evaluates probability also changes with context.

It has been apparent to behavioral ecologists interested in risk-sensitive foraging theory (RSFT) that dynamic changes in risk attitudes occur across time within individual foraging animals (Caraco, 1981; Hayden and Platt, 2009; Kacelnik and Bateson, 1997; McNamara and Houston, 1992; Real and Caraco, 1986). For example, during the day, warm-blooded animals pursue safe but small-sized prey items—those that they probably will be successful in obtaining but which have a low food value. However, they may pursue choices that are riskier but have a higher value as evening approaches and foraging opportunities for the day decrease. This is particularly the case if their metabolic resources are low or if they need to gather enough food to meet a metabolic target to survive a cold night. In such circumstances, pursuing a safe option associated with a probable but small magnitude of food is, by evening, of little long-term value, because it will not be sufficient to guarantee the animal’s survival through the night. Instead the animal should be biased toward riskier options associated with high magnitudes of food items, even if they have a lower probability of success. The animal’s attitude to probabilities is, therefore, a function of its momentary resource budget and its longer term targets.

From this ecological perspective, decisions are viewed as occurring within sequences, and there is the possibility of adapting the decision-making strategy later within such a sequence depending on the outcomes of initial decisions. Therefore, when navigating an environment via a sequence of decisions, riskier choices can be seen as part of a particular strategy, influenced by past experiences and future prospects.

The first aim of the study was to test whether dynamic changes in decision strategy occur in humans as they make a series of decisions and to see whether they depended on a person’s current resources as well as longer term targets. Thus, while our approach borrows from RSFT, it differs from most accounts of “risk” and gambling prevalent in cognitive neuroscience because it recognizes that different use of probability—effectively different decision-making strategies—may be optimal in different contexts. Some contexts, such as the prospect of the cold night for the foraging animal described earlier, can be thought of as exerting a pressure to assume a more risky decision-making strategy. We refer to this contextual influence as risk pressure. Note that we use the term “risk” in the sense it is most commonly used (to refer to a choice’s probability to yield no gain or a loss) rather than in the way it is sometimes used in neuroeconomics (to refer to the outcome variance of a choice) (Platt and Huettel, 2008; Preuschoff et al., 2006; Rushworth and Behrens, 2008).

**Figure 1:**
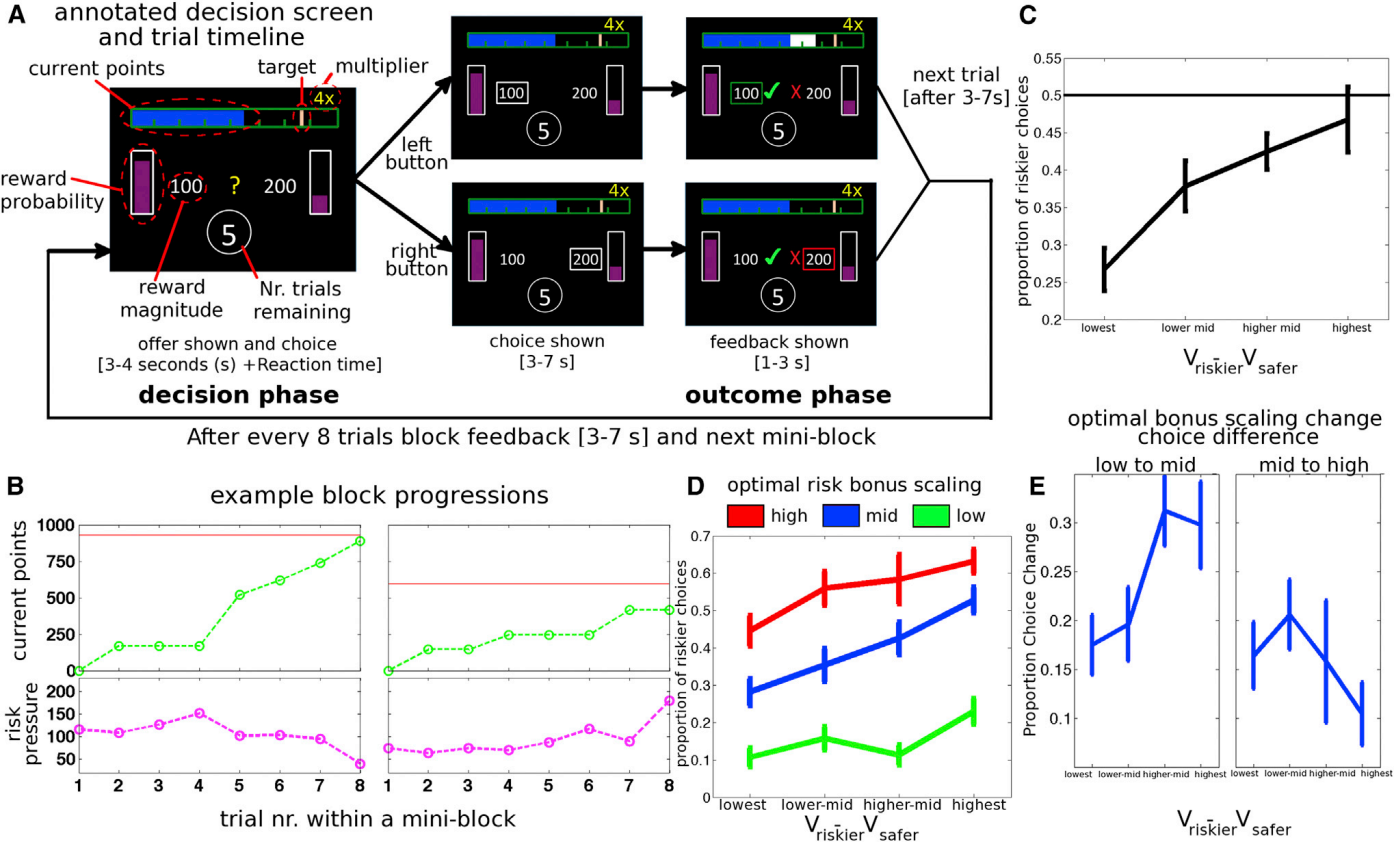
Behavioral Task and Results (A) Trial timeline: at the start of trials, subjects were presented with choices on the left and right of the screen. Each option was composed of a reward probability (height of purple bar fill) and magnitude (number next to each bar). This was followed by a choice cue (yellow question mark) that instructed subjects to choose. Subjects chose between a more probable low-magnitude option (safer option) and a less probable high-magnitude option (riskier option) on each trial. After responding, their choice was highlighted with a white frame, and feedback was shown for both options (both the chosen option and the alternative). If the choice was rewarded, then points were added to the blue “current points” bar at the top of the screen (white bar indicated added points), progressing it further toward the target. The number of trials remaining in the block was indicated in the circle at the bottom of the screen. After each trial, the number of remaining trials was reduced by one. The target turned white if it was reached. (B) Two examples of progressions through a miniblock. Points accumulated are shown in green, with target level in red (upper panel) and the resulting risk pressure in magenta (lower panel). In the first example, the target was relatively high, and the risk pressure is highest before a big win after the fourth decision, when the subject selected the less likely but more valuable riskier option. In the second example, the pressure is lower at first but increases after a series of losses until it actually exceeds the risk pressure experienced in the other block. nr., number. (C) Overall proportion of riskier choices as a function of increasing relative value of riskier choice (V_riskier_ − V_safer_). (D) Overall proportion of riskier choices split by optimal risk bonus scaling and binned by increasing relative value of the riskier choice (V_riskier_ − V_safer_). (E) Differences in proportion of riskier choices (left) between low-level and midlevel (green and blue, respectively, in D) and (right) between midlevel and high-level (blue and red, respectively, in D) optimal risk bonus scaling, illustrating how changes in optimal risk bonus scaling are associated with increased frequencies of riskier choices. Additionally, for the first change, choices with large V_riskier_ − V_safer_ value differences are affected more, whereas, for the second change, the more difficult decisions involving lower V_riskier_ − V_safer_ value differences are more affected. All error bars represent means ± SEM. See also Figures S1 and S2.

We simulated the situation of a foraging animal pursuing an imperative longer term reward target by asking subjects to try and repeatedly collect a target number of points over a miniblock of eight decision trials. Subjects chose between safer “high probability” options and riskier, but high-magnitude, options (Figure 1A). Reaching the target meant that subjects kept the points they won in that block, but failure to reach the target meant that all points in that eight-trial block were lost. Thus, the subjects accumulated resources in terms of points, and with every decision, their foraging opportunities, in terms of trials left in the block, decreased. It is important to note that the safer choice normally had, on average, a higher value (on six of eight times, it had the higher expected value [probability × magnitude] and, in general, it was preferred by participants). However, if a subject takes into account the sequential structure of the task as well as the contextual factors—i.e., the target level, their current level of resources, and the number of trials left—then it should motivate them to take the riskier choice instead. This is because, even if it is successful, the safer choice sometimes yields insufficient points to reach the target.

Our analysis focused on relating decisions and brain activity recorded with fMRI to two types of variables. The first type concerned specific decisions that participants made and the choice values that motivated those decisions. This part of the analysis often concerned the relative values of riskier and safer choices (V = value; V_riskier_ − V_safer_). In the past, relative value signals have been used to identify neural mechanisms of decision making (Boorman et al., 2009; Camille et al., 2011; De Martino et al., 2013; Fellows, 2011; FitzGerald et al., 2009; Hunt et al., 2012; Kolling et al., 2012; Lim et al., 2011; Noonan et al., 2010; Philiastides et al., 2010; Wunderlich et al., 2012). The second type of variable focused on the gradually changing context as participants moved through the block. For this, we estimated three key parametrically varying quantities. First, trial number indexed how far through the block the subject had progressed. Second, risk pressure was the difference between the subject’s current resources and the imperative target scaled by the remaining foraging opportunities (Equation 1; Figure 1B). Risk pressure should lead to a contextual modification of the options’ values. Using a model, we formalized the amount of optimal modification in a given trial through the third key term: risk bonus (Equation 6), the degree to which risk pressure should optimally bias a person away from the safer choice, given the current offers’ magnitudes and probabilities, as well as future decision opportunities. Further information about the regressors is provided in Experimental Procedures and in the Supplemental Experimental Procedures available online. All the regressors used in a given whole-brain analysis shared less than 25% of their variance, making it possible to identify variance in the fMRI-recorded activity related to each (Figure S2). The fMRI analysis focused on two frontal areas, ventromedial prefrontal cortex (vmPFC) and dorsal anterior cingulate cortex (dACC), implicated in decision making (Hare et al., 2011; Kolling et al., 2012).

## Results

### Behavioral Results

Subjects had a baseline tendency toward risk aversion, but they took more risky choices as risk pressure increased. This is apparent when trials are binned into four levels according to the V_riskier_ − V_safer_ value difference and the frequency of riskier choices is plotted (Figure 1C). Overall, participants were less likely to take riskier choices at all levels of V_riskier_ − V_safer_ difference, but the effect was smaller when the value difference for V_riskier_ − V_safer_ was larger.

The frequency of riskier choices can be examined not just as a function of the V_riskier_ − V_safer_ value difference but also as a function of optimal risk bonus scaling, which is one of the parameters derived from our model that expresses the approximately optimal degree to which participants should be biased toward riskier choices as risk pressure increases independent of the specific options presented in the trial (Figure 1D). Three equally sized bins of trials were created using the optimal risk bonus scaling factor for a trial. Within each level of optimal risk bonus scaling, we examined the effect of the V_riskier_ − V_safer_ value difference. Participants took more risky choices when V_riskier_ − V_safer_ value difference was larger, even when the optimal risk bonus scaling was lowest. On trials with little or no optimal risk bonus scaling, participants did not, on average, prefer riskier choices, even when the V_riskier_ − V_safer_ value difference was high (there was no significant preference with a one-tailed t test against 0.5; see Figure S1). However, when optimal risk bonus scaling was high, participants began taking more risky choices, even when the V_riskier_ − V_safer_ value difference was in the lower midrange. A change in optimal risk bonus scaling from low levels to midlevels (Figure 1E, left) and from midlevels to high levels (Figure 1E, right) is associated with an increased frequency of taking riskier choices. In the first case, decisions with large V_riskier_ − V_safer_ value differences are affected, whereas in the second case, the more difficult decisions involving lower V_riskier_ − V_safer_ value differences are more affected.

**Figure 2:**
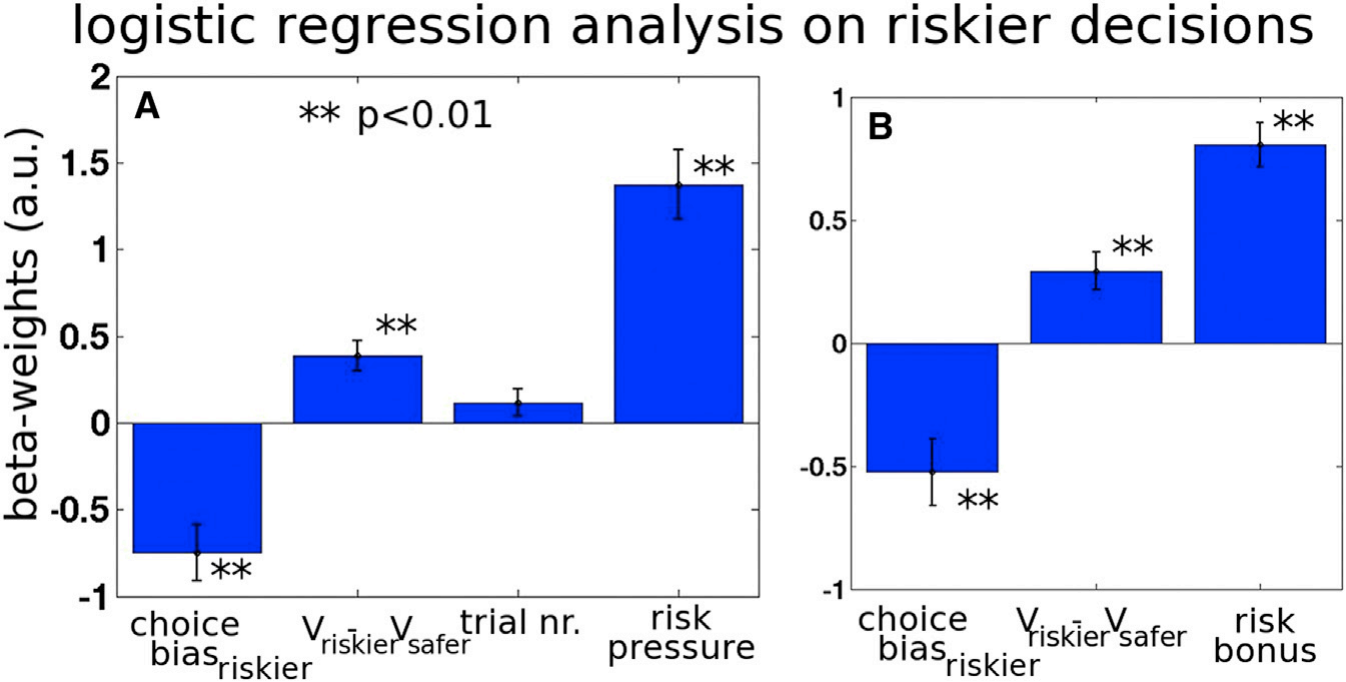
Logistic Regression of Riskier Choices against Parameters Defining Each Decision (A) GLM incorporating relative value of riskier choice (V_riskier_ − V_safer_), number of trials already performed in the current block (trial number, nr.), and risk pressure. Increases in both V_riskier_ − V_safer_ and risk pressure were associated with significant increased riskier choices. The constant term from the GLM, however, indicates a bias against riskier choices (left bar with negative value). a.u., arbitrary units. (B) An alternative analysis used risk bonus (reflecting the model-based impact of the current risk pressure on V_riskier_ − V_safer_); again, increases in this term were associated with significant increases in riskier choices. All error bars represent means ± SEM. See also Figures S3 and S4.

We tested whether the frequency of riskier choices was simply driven by V_riskier_ − V_safer_ value differences or whether it also reflected the risk pressure associated with the context in which the decision occurred, using a logistic regression analysis (see the Behavioral Analysis section in Experimental Procedures). The V_riskier_ − V_safer_ value difference exerted a significant influence, t(17) = 4.48, p < 0.001, but this is obviously expected, given that our estimates of the subjects’ values are based on their choices (Equation 2). What is important to note, however, is that it was not sufficient to explain choices; risk pressure exerted an additional effect, t(17) = 6.88, p < 0.001 (Figure 2A). An alternative logistic regression looked at riskier choices as a function of the risk bonus on each trial (this term expresses how the relative value of the riskier option as opposed to the safer option changes as a function of risk pressure and the option’s specific magnitudes and probabilities; Equation 5). The risk bonus on a trial exerted a significant impact on riskier choice frequency, t(17) = 9.03, p < 0.001 (Figure 2B). Note that both analyses included a negative constant term (negative-going bar on the left side of Figures 2A and 2B, in form of the intercept of the regression model); this means that subjects were biased against riskier choices and that their default approach was to take safer choices, although they did so less when the V_riskier_ − V_safer_ value difference or risk pressure was higher. Figure S3A shows the results of another logistic regression that incorporates both the regressors shown in Figures 2A and 2B.

Another way to examine how participants shifted away from a baseline tendency to risk aversion is to compare their behavior to the predictions of our model, which, as already noted, makes decisions that are close to optimal. Participants were more likely to make model-conforming safer choices than they were to make model-conforming riskier choices (Figure S4B). However, riskier choices were still more likely than not to conform to model predictions. This means that, even though participants were not completely optimal, they integrated over choice value and contextual factors in a way predicted by our model, with a slight overall bias against the riskier option.

## Results

### Contextual Modification of Value

**Figure 3:**
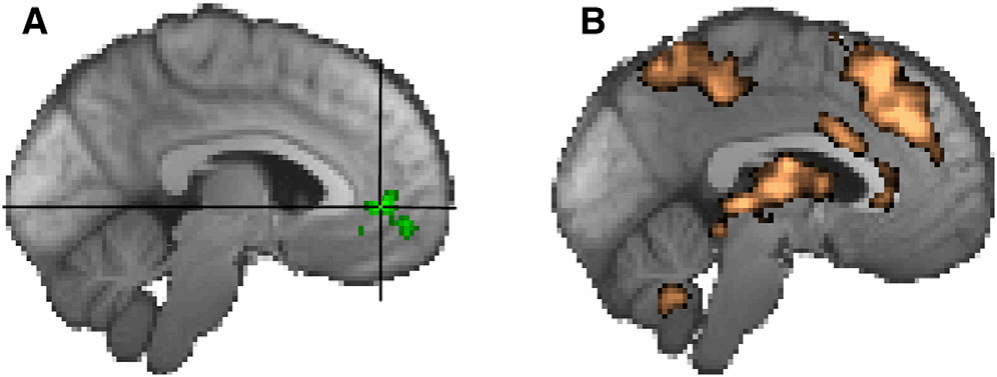
Decision Making in the Absence of Risk Pressure and Task Progression Effects (A) Decreasing risk bonus was associated with increased vmPFC activity. The impact was present, regardless of subjects’ choices. (B) Activity increases in dACC and elsewhere during the decision phase as number of trials remaining decreased.

To look at the impact of context, we split all trials into those where the context meant that there was a risk bonus and those where there was none (see Supplemental Experimental Procedures). First, we looked at the main effect of the risk bonus, in other words, we looked at the model-based modification of each trial’s option values away from the default safer choice in favor of the riskier choice as a result of risk pressure. We observed a relative decrease in vmPFC activity as risk bonus increased that was independent of which choice, riskier or safer, subjects ultimately made (Figure 3A). In other words, vmPFC activity is negatively related to the risk bonus. Beyond this choice-independent decrease, we were unable to find any choice-related value signals, either “raw” ones (Equation 2) or contextually modified ones (Equations 3, 4, and 5) (such as an absolute or relative chosen value signal). This is in stark contrast to most other studies that have suggested that vmPFC codes the value or relative value of potential or attended choices (Boorman et al., 2009; De Martino et al., 2013; FitzGerald et al., 2009; Hunt et al., 2012; Kolling et al., 2012; Lim et al., 2011; Philiastides et al., 2010; Wunderlich et al., 2012). In summary, while vmPFC may normally track choice values during decision making, it does not do so in the current paradigm, in which both immediate value and current risk bonus had to be integrated to make appropriate choices. Instead, vmPFC’s activity decreased if the context meant that there was a risk bonus, and subjects increasingly biased their decisions toward the riskier choice and away from the default of taking the safer choice.

### Sequential Progression and the Generation of Riskier Choice Decisions

**Figure 4:**
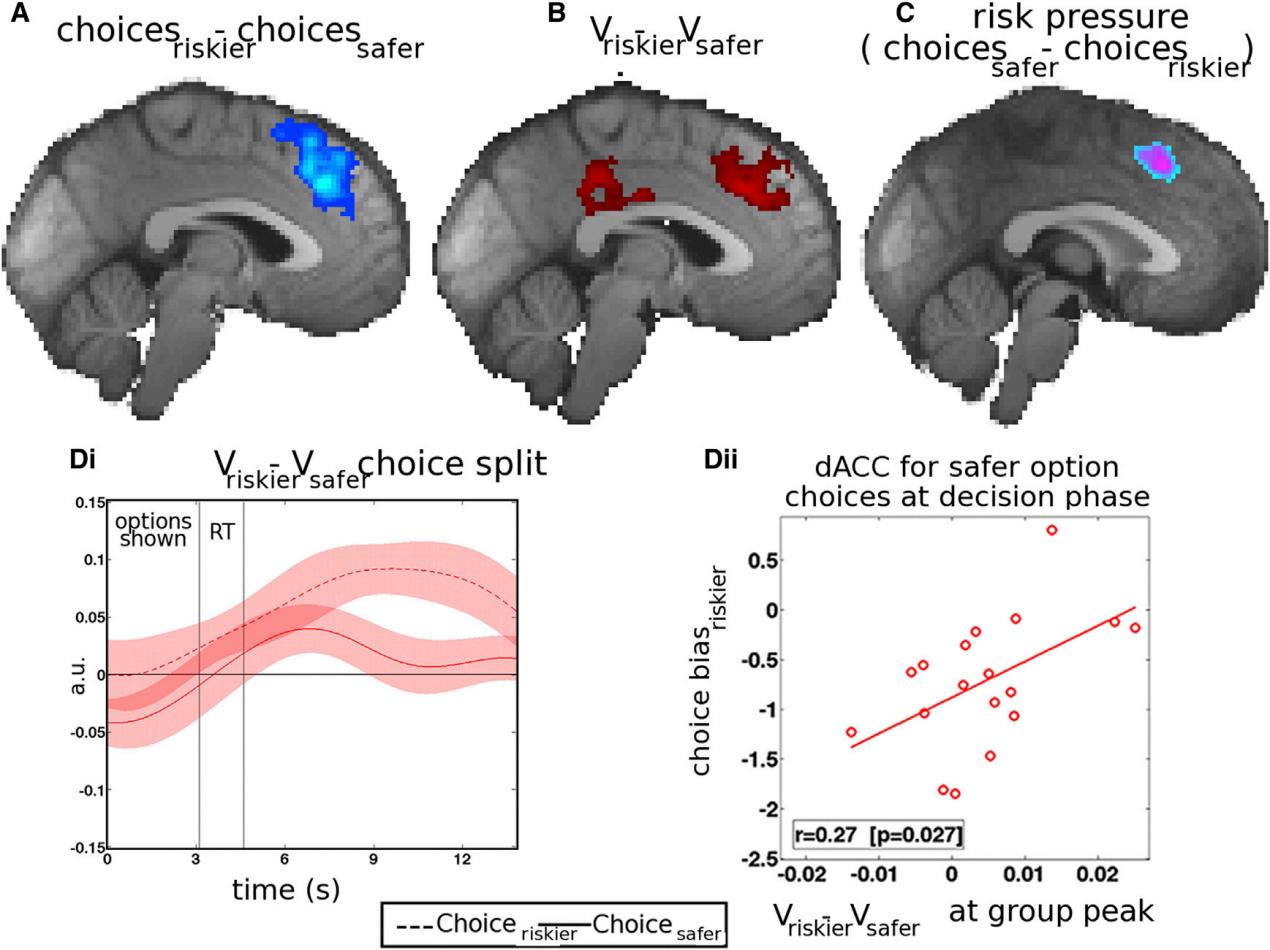
dACC at Decision I (A) When just the main effect of choice is considered, then activity in dACC and adjacent dorsomedial frontal cortex increased for riskier as opposed to safer choices. (B) Again, the same dACC region and adjacent dorsomedial frontal cortex exhibited increased activity as a function of the relative value of riskier choices (V_riskier_ − V_safer_). (C) dACC activity during the decision phase increased as a function of risk pressure when subjects did not succumb to it and instead made riskier rather than safer choices. (D) In (i), the dACC group time course of the V_riskier_ − V_safer_ effect is shown separately for riskier (continuous line) and safer choices (dotted line). In (ii), subjects who were less biased against riskier choices exhibited a higher dACC V_riskier_ − V_safer_ effect at the peak of the group time course. a.u., arbitrary units; RT, reaction time. See also Figure S5.

**Figure 5:**
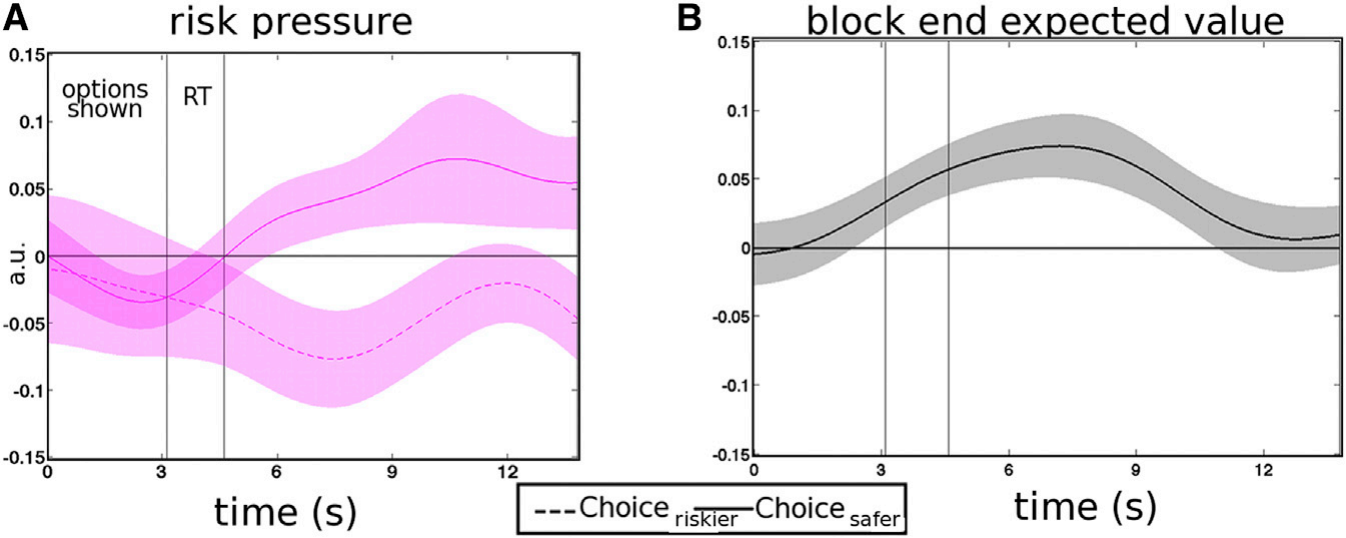
dACC at Decision II (A) The risk pressure signal in dACC increased when subjects did not act in accordance with it, but it had a negative effect when subjects did act in accordance with risk pressure and took the riskier option. a.u., arbitrary units. (B) Independent of choice (riskier or safer), higher expected value at the end of the block, as estimated using our model, was related to increased dACC activity. See also Figure S6.

Progression through the eight-trial miniblocks had a strong impact on activity in dACC and other regions (Figure 3B). Crucially, in addition to this effect, dACC was sensitive to another piece of information more directly related to the decision strategy that subjects used; its activity was correlated with risk pressure, the average points per trial needed to reach the target (Figure 4C). However, the effect of risk pressure on dACC activity reversed depending on choice. A positive effect of risk pressure on dACC activity was apparent when subjects chose the safer option, whereas a negative effect was apparent when subjects chose the riskier option. In other words, dACC activity increased with increasing risk pressure when choices went against the prevailing risk pressure but decreased with increasing risk pressure when subjects chose in agreement with risk pressure (Figures 4C and 5A).

The dACC risk pressure signal cannot be explained away as a signal-indexing approach toward a reward that might be delivered at the end of the block (Croxson et al., 2009; Shidara and Richmond, 2002), because progress through the sequence of trials itself was present as a separate regressor in the general linear model (GLM) and associated with an independent effect on dACC activity (this is the effect already shown; Figure 3B). The risk pressure signal cannot be explained away as a consequence of differing average reward expectations associated with different target levels because the use of a “multiplier” procedure (see the Experimental Task section in Experimental Procedures) ensured that average reward expectations were the same at the beginning of a block regardless of the target. It is, however, the case that expectations about the reward that would be received at the end of the block (as opposed to just after the current trial within the block) began to diverge as soon as participants began to make choices and were either lucky or unlucky. However, when we included an additional term in the GLM indexing the expected value of the reward at the end of the block we found that it had an independent effect on dACC activity (Figure 5B). No similar signal was observed in vmPFC (Figure S6). In summary, dACC exhibited a number of signals related to progress through the sequence of decisions, the expected reward at the end of the sequence, and a risk pressure signal indexing the need to take riskier choices as a function of contextual factors (accumulated resources, target, and remaining foraging opportunities). The risk pressure signal flipped with the decision strategy that subjects pursued (safer versus riskier); it was positive when subjects needed to change their behavior and switch to riskier choices as opposed to the default safer choice.

In addition to these contextual effects, the same dACC region also exhibited activity that was tied to specific patterns of choice and choice valuation. dACC activity was higher in decisions in which the riskier rather than the safer choice was taken (choice_riskier_ − choice_safer_; Figure 4A). Not only did the main effect of the choice_riskier_ − choice_safer_ difference activate dACC, but so did the relative value of the riskier choice (V_riskier_ − V_safer_; Figure 4B). Moreover, the signal encoding the V_riskier_ − V_safer_ value difference was stronger on trials on which subjects actually took the riskier choice, although it was also present when subjects took the safer choice (Figure 4Di). Individual variation in the V_riskier_ − V_safer_ signal size at the group peak coordinate in dACC when taking the safer choice was related to how frequently subjects took the riskier choice (Figure 4Dii), suggesting that variation in this aspect of dACC activity is intimately related to decision making.

Activity increases related to the choice_riskier_ − choice_safer_ contrast were also apparent in the inferior frontal gyri (IFG) and frontal operculum (Table S1), while the V_riskier_ − V_safer_ contrast was also associated with activity in posterior cingulate cortex (PCC) (Figure 4B; Figure S5) and dorsolateral prefrontal cortex (dlPFC). We propose an explanation of IFG and PCC activity in a later section. In summary, one region—dACC—encoded five features of the task: (1) the expected reward at the end of the sequence of decisions, (2) progress through the sequence of decisions, (3) risk pressure, (4) taking riskier choices but not taking safer choices, and (5) the relative value of the riskier choice versus the safer choice. The time course analyses shown in Figures 4D and 5 are all from the same region of interest with Montreal Neurological Institute coordinates x = −2, y = 28, and z = 36.

Although further experiments are needed to determine quite why the impact of risk pressure on dACC activity changed depending on whether subjects acted in accordance with it or not, it is worth considering that it may reflect the operation of an evidence accumulation process to threshold that finally results in a riskier choice being taken. This would be consistent with the observation that actually taking a riskier choice activates dACC (Figure 4A), as does the evidence advocating such a choice (V_riskier_ − V_safer_; Figure 4B). If an accumulation process is taking place before riskier choices are generated, then it seems that risk pressure increases such activity (Figure 4C). However, once such a process has hit its bounds, triggering the taking of a riskier choice, further activity increases related to the risk pressure are not observed. Although there is evidence for the operation of accumulation processes in dACC (Hayden et al., 2011; Kolling et al., 2012), further experiments are needed to determine whether risk pressure is contributing to such a process.

**Figure 6:**
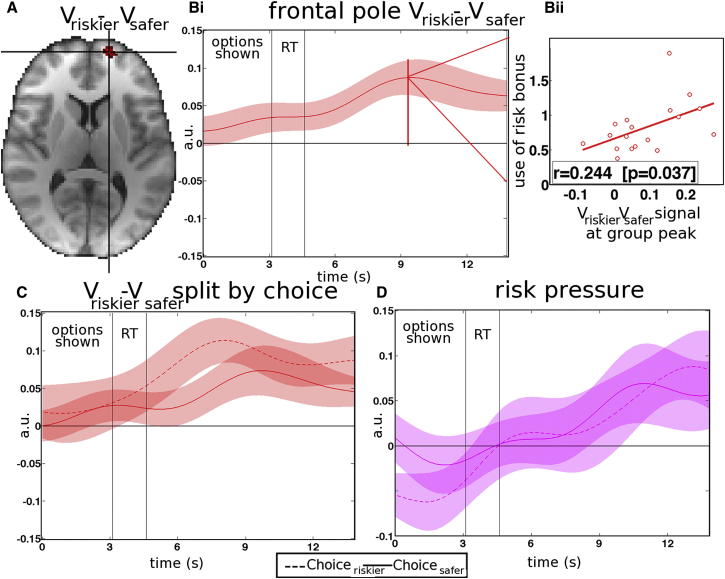
FP at Decision (A) The FPl carried a V_riskier_ − V_safer_ signal regardless of which choice subjects ultimately made. (B) In (i), the group time course of the V_riskier_ − V_safer_ signal (regardless of whether riskier or safer decisions were made) is shown. In (ii), individual subject V_riskier_ − V_safer_ signal effect sizes at the peak of the group time course predicted individual behavioral sensitivity to risk bonus. a.u., arbitrary units. (C) The V_riskier_ − V_safer_ signal was present in FPl both for riskier choices (continuous line) and safer choices (dotted line). (D) Risk pressure activated PFl similarly for riskier choices (continuous line) and safer choices (dotted line).

In the past, another region, the lateral frontal pole (FPl), has been associated with tracking the values of alternative courses of action (Boorman et al., 2009, 2011). FPl activity also increased as a function of V_riskier_ − V_safer_ value difference (Figure 6), and individual differences’ signal strength were related to individual differences in the degree to which subjects modulated their behavior according to the model-based risk bonus (Figure 6Bii). Unlike in dACC, FPl signals tracking risk pressure and V_riskier_ − V_safer_ value difference were apparent regardless of which choice, riskier or safer, subjects took (Figures 6C and 6D). In other words, FPl provides a constant signal, regardless of current choice type, of how necessary it is to adjust choice strategy away from the default safer choice and toward the riskier choice in the face of risk pressure.

### Outcome-Related Signals

So far, we have shown that dACC is more active when a riskier choice, as opposed to a safer choice, is made (Figure 4A) and that dACC activity reflects the relative value of riskier choices (Figure 4B) and risk pressure (Figure 4C). Next, we consider whether dACC also contains signals related to evaluation of the success of riskier choices when their outcomes are revealed. Subjects can update their estimate of risk pressure or the likelihood that they will reach the target when they see the outcome of their choice. Therefore, we tested whether dACC activity was related to changes in risk pressure at the time of outcome presentation.

**Figure 7:**
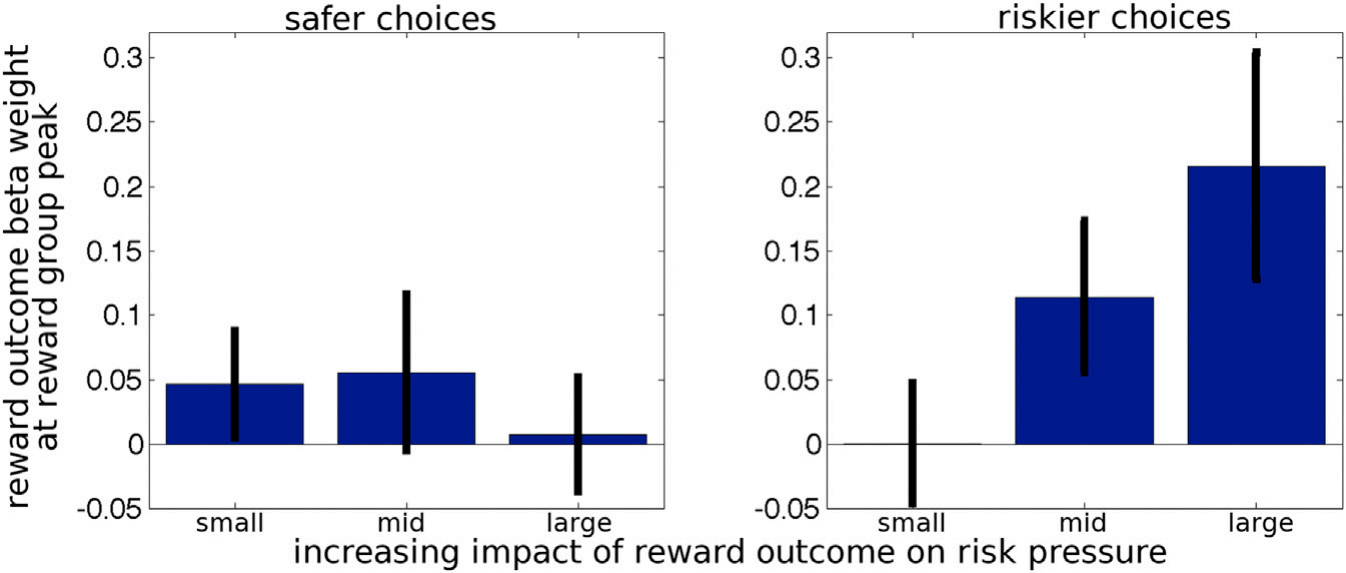
The Effect of Outcomes on Activity in the dACC after Riskier or Safer Choices The effect of outcomes is not only dependent on choice (riskier or safer) but also on how much impact they have on future risk pressure (greater on the right than on the left of each panel).

To do this, we plotted the effect of decision outcome on dACC activity after safer and after riskier choices. In addition, we also binned the outcome effects according to three levels of the change they caused to risk pressure. In other words, we examined the effect of two factors, choice type (riskier versus safer) and the size of impact of outcome on risk pressure (three levels: low, medium, and high). There was a significant interaction between the two factors on outcome-related dACC activity, F(2, 34) = 3.417, p = 0.044. As the outcome’s impact on risk pressure increased, so did the outcome’s impact on dACC activity, but this was only the case when riskier choices were taken (Figure 7, right). After safer choices (Figure 7, left), there was no increase in the impact an outcome had on risk pressure (in fact, if anything, there was a slight decrease). The results remained the same even after controlling for the expected value of the whole block, F(2, 34) = 4.352, p = 0.021, and outcome surprise, F(2, 34) = 3.848, p = 0.031. At the time of outcomes, dACC is not only simply encoding prediction errors in value (Jocham et al., 2009; Kennerley et al., 2011; Matsumoto et al., 2007) but also the impact that riskier choices have on reducing risk pressure.

### Functional Connectivity and Networks of Choice

A large body of work has implicated vmPFC in reward-guided decision making, but it was deactivated in the current experiment when the subject’s context meant that the default safer choice should not be taken and the riskier choice should be taken instead (risk bonus effect; Figure 3). By contrast, dACC activity increased with risk pressure and was greatest when subjects chose the riskier choice (Figure 4). Therefore, it seems that the two frontal brain regions, vmPFC and dACC, may mediate decisions in different situations. If there are two systems competing to control behavior, then it is not clear how the competition is resolved or if there is any critical area that mediates both types of decisions.

One region that may be a nexus for both types of decision modes is the PCC. In many neuroimaging studies, it carries a value difference signal like that seen in the vmPFC (Boorman et al., 2013; FitzGerald et al., 2009; Kolling et al., 2012). However, a series of single-neuron recording studies have emphasized the similarities between the parameters that both it and dACC encode (Pearson et al., 2011), and in the current study, it, like dACC, was sensitive to the relative value of riskier choices (V_riskier_ − V_safer_) (Figure 4B). The PCC region that was active in this contrast probably includes areas 31 and 23, but it also includes the caudal cingulate motor areas that lie in the cingulate sulcus at the point of its inflection into its marginal ramus (Amiez and Petrides, 2012; Beckmann et al., 2009). In macaques, the caudal cingulate motor area projects to both the primary motor cortex and ventral horn of the spinal cord (Dum and Strick, 1996), so it may be involved in making the movement needed for implementing a particular choice. In macaques, it is connected to the dACC, vmPFC, and adjacent parts of PCC (Parvizi et al., 2006; Van Hoesen et al., 1993), so it is, therefore, a region through which vmPFC, dACC, and PCC might interact and influence action movement selection.

**Figure 8:**
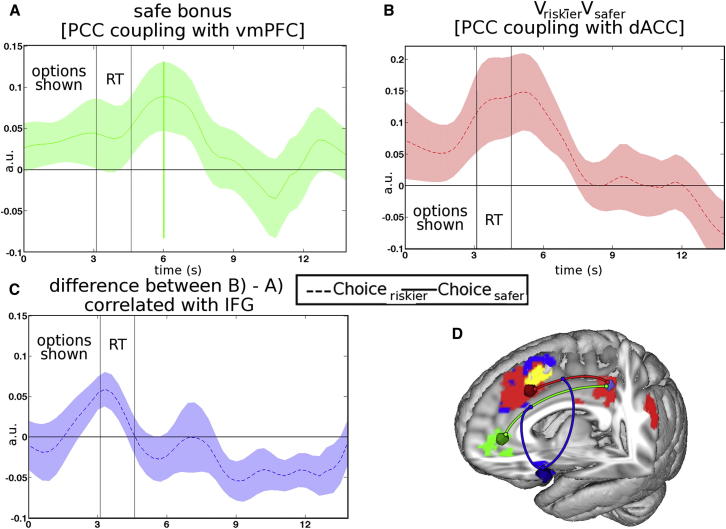
PPI Analyses Demonstrated vmPFC and dACC Interactions with PCC during Different Types of Decisions and the Relationship with IFG Activity (A) Time course illustrating PPI between PCC and vmPFC as a function of decreasing risk bonus on trials when the safer option was taken. a.u., arbitrary units. (B) Time course of PPI between PCC and dACC as a function of V_riskier_ − V_safer_ value difference on trials on which the riskier option was chosen (dotted line). (C) Time course of a PPI on PCC, using the IFG signal and the different effects of vmPFC and dACC, the two regressors from (B) and (A), respectively. (D) Illustration of effects in (A)–(C). The PCC couples with dACC and vmPFC during decisions in which the riskier option (red) and safer option (green) were taken, respectively. Left IFG may regulate PCC’s interactions with the vmPFC and dACC by increasing the relative degree of coupling to the former as opposed to the latter during riskier choices. See also Figures S7 and S8.

We conducted a psychophysiological interaction (PPI) test of whether vmPFC and dACC activities were coupled with PCC activity in different ways as a function of choice (riskier or safer) and their relative values (V_riskier_ − V_safer_). There was greater coupling between dACC and PCC as a function of V_riskier_ − V_safer_ value difference but only when the riskier choice was chosen (Figure 8B). In other words, PCC’s coupling with dACC increases as a function of the decision variable, V_riskier_ − V_safer_ value difference, which predisposes participants to take riskier choices (Figures 1 and 2) and which influences dACC activity (Figure 4). By contrast, vmPFC was more coupled with PCC when the default safer choice was taken and as a function of risk bonus being low (Figure 8A). In other words, PCC’s coupling with vmPFC increased in inverse relationship with the decision variable risk bonus. The inverse of risk bonus was associated both with lower vmPFC activity (Figure 3A) and with higher frequencies of taking the default safer option (Figures 1 and 2). PCC carries signals that are more similar to either vmPFC or dACC, depending on the prevailing context at the time of each decision and depending on the choice that subjects actually took (for the coupling pattern of the ventral striatum, see Figure S7).

Finally, we looked for evidence of a brain area that might resolve competition between dACC and vmPFC and determine which one couples with PCC. We focused on IFG because we had noticed that its activity changed with choice in the current experiment, even though it did not carry a value signal (discussed earlier), and because it has been argued that it or adjacent regions exert a regulatory influence over vmPFC activity in other situations (Baumgartner et al., 2011; Hare et al., 2009). We carried out a further PPI analysis that, once again, tested vmPFC-PCC and dACC-PCC coupling, but this time, we examined vmPFC-PCC and dACC-PCC coupling as a function of IFG activity. PCC’s coupling with dACC versus vmPFC was related to IFG activity when the riskier choice was chosen (Figure 8C). In other words, with increasing IFG activity, the relative strength of dACC-PCC coupling increased (which was also, as described earlier, a function of the V_riskier_ − V_safer_ value difference) as opposed to vmPFC-PCC coupling (which was also, as described earlier, a function of low risk bonus). Such a pattern of results is consistent with a controlling function for IFG, not just of activity in other brain regions but also of the interconnectivity between other brain regions. A clear demonstration of the causal direction of effects, however, would require showing that IFG disruption affected the coupling patterns.

## Discussion

### Dynamic Changes in the Use of Probability

Instead of assuming that attitudes to probabilities reflect stable individual differences, a behavioral-ecological approach to decision making suggests that animals should adapt decision-making strategies as a function of their current resources, resource targets, and the opportunities that remain for foraging (Caraco, 1981; Hayden and Platt, 2009; Kacelnik and Bateson, 1997; McNamara and Houston, 1992; Real and Caraco, 1986). We argue that these factors can be integrated to determine the current risk pressure—the degree to which it might be adaptive to adjust decision making toward pursuit of low probability but potentially large reward magnitude outcomes. The combination of risk pressure with the precise values of the specific options that might be chosen in a given decision determine a risk bonus—an increase in value that accrues to the low probability but potentially large magnitude option in a decision. We designed a decision-making task for humans (Figures 1A and 1B) that manipulated these factors, changing resource levels, target levels, and opportunities for further foraging. Human subjects were sensitive to risk pressure and the risk bonus; increases in each factor led to more frequent riskier choices (Figures 1 and 2). Although we think that our approach of adding a risk bonus to the values of choices that are made in the context of risk pressure provides an intuitive way to think about how decision-making strategies can be rapidly updated, there are, nevertheless, links between several of the concepts used in our approach and those that can be derived from a reinforcement learning-based approach (Supplemental Experimental Procedures).

We demonstrated a neural correlate of continuous tracking of changing context that, in turn, impacted on evaluation of specific choices. The approach is, however, complementary to normative approaches that have described how preferences are expressed and updated. There is a link to previous studies that have shown that subjects often have biases toward certain decisions and that activity in some brain regions is associated with taking decisions that do not conform with the default strategy (Venkatraman et al., 2009a, 2009b).

Individual differences in risk-taking behavior may, in extreme cases, be associated with pathological gambling (Clark and Limbrick-Oldfield, 2013). While pathological gambling may be linked with a baseline change in risk proneness/aversion, our results raise the possibility of a link with individual differences in how decisions are influenced by context. An approach focusing on changing sensitivity to contextual factors such as risk pressure may elucidate aspects of developmental change in risky behavior (Blakemore and Robbins, 2012; Paulsen et al., 2012). Assaying response strategies with low likelihoods of success but with the potential for delivering great gains may be imperative at some points in adolescence.

### Neural Systems for Decision Making

VmPFC and dACC might constitute two distinct decision-making systems rather than components of a single serial system for decision making (Boorman et al., 2013; Kolling et al., 2012; Rushworth et al., 2012). There was evidence that vmPFC and dACC acted in independent, or even opposite, ways in the current study.

Although there has been particular interest in the role that vmPFC plays in valuation and decision making (Boorman et al., 2009; Camille et al., 2011; De Martino et al., 2013; Fellows, 2011; FitzGerald et al., 2009; Hunt et al., 2012; Kolling et al., 2012; Lim et al., 2011; Noonan et al., 2010; Philiastides et al., 2010; Wunderlich et al., 2012), vmPFC did not mediate the influence of the contextual variable of risk pressure on decision making. Instead, vmPFC became less active as risk bonus increased (Figure 3A). Both lesion and neuroimaging evidence suggest that, in addition to its role in valuation and decision making, vmPFC mediates the repetition of a previously successful choice or the taking of a default choice (Boorman et al., 2013; Noonan et al., 2012, 2010), and the pattern of activity recorded in vmPFC suggests that it was similarly concerned with default responses in the present task. This interpretation is suggested by the following observations. On average, subjects were risk averse and defaulted to taking the safer choice. This was most true on trials in which the risk pressure was low (Figures 1 and 2), and it was on just such trials that vmPFC activity was greatest (Figure 3A). Note that, in this task, default choices occur when decision making is less constrained by context.

Instead of vmPFC, both dACC and FPl were preeminent in tracking the risk pressure afforded by the evolving decision context (Figures 4, 5, 6, and 7). FPl and dACC have been coactivated in other studies (Boorman et al., 2011; Daw et al., 2006); together, they constitute another neural system important for decision making. In macaques, frontal pole (FP) and dACC are monosynaptically interconnected (Petrides and Pandya, 2007). There is evidence that FPl, unlike medial FP, is only found in humans and not in other primates but that it remains interconnected with dACC (Neubert et al., 2014).

In FPl, signals indicating both risk pressure and V_riskier_ − V_safer_ value difference were present, regardless of the choice (riskier or safer) subjects took. By contrast, in dACC, both signals changed as a function of choice, and the taking of riskier choices was associated with additional activity (Figures 4 and 5). These observations suggest that dACC was more closely related to the actual decision to take a specific riskier option, while FPl had a more consistent role in tracking the contextual variables that guided decisions. Individual variation in the sizes of both FPl and dACC signals were predictive of subjects’ sensitivities to the risk bonus and their predispositions to make riskier choices (Figures 4Di and 6Bii). Individual variation in the V_riskier_ − V_safer_ signal in dACC, when the safer choice was taken, predicted how frequently subjects rejected the default safer choice and took the alternative riskier option. This is consistent with the idea that, when one course of action is being pursued or is the apparent default course of action, dACC is tracking the value of switching to an alternative (Kolling et al., 2012; Rushworth et al., 2012). In a previous study, dACC also encoded the relative value of switching away from the current default choice to explore a foraging environment (Kolling et al., 2012). An “inverse value difference” signal is often seen in dACC (Kolling et al., 2012; Rushworth et al., 2012); when a decision is being made, dACC activity increases as the value of the choice not taken increases, and it decreases as the value of the choice that is taken increases. This signal is opposite to the one seen in vmPFC. One simple interpretation of the dACC inverse value signal is that it is encoding the value of switching away from the current choice to an alternative one.

So far, we have focused on dACC signals that are recorded at the time when decisions are made, but dACC activity is also observed subsequently at the time of decision outcomes. Outcome-related dACC signals can also be interpreted in a similar framework and related to the need to switch away from a current choice and to explore alternatives (Hayden et al., 2009, 2011; Quilodran et al., 2008).

A notable feature of dACC activity in the present study was that, unlike vmPFC activity, it reflected the longer term value of a course of action, progress through the sequence of decisions, and the evolving level of risk pressure (Figures 3B, 4C, and 5). Boorman and colleagues (2013) have also argued that dACC reflects the longer term value of a choice and not just its value at the time of the current decision that is being taken. Not only does dACC carry signals related to the longer term and contextually modified value of a choice, but it also encodes the approximate value of a number of potential alternative courses of action (Kolling et al., 2012). By contrast, vmPFC is more concerned with the valuation of specific aspects of specific choices. Value-related activity in vmPFC is most prominent when the choices’ values are determined by multiple attributes and when it is necessary to identify the attribute currently most relevant for guiding a choice (Fellows, 2006; Hunt et al., 2012).

One prominent account of dACC function has emphasized its role in detecting response conflict (Botvinick, 2007). Although some features of the dACC results are consistent with the response conflict account, other features, such as the value difference signal (V_riskier_-V_safer_) in dACC are not easy to interpret within the framework offered by the conflict account; the dACC V_riskier_-V_safer_ signal encodes the relative value of the riskier choice as opposed to the safer choice but it was stronger when that very same choice, the riskier choice, was being made and when, because of its relatively greater value, the decision should have been relatively easy to take.

### PCC as a Final Common Pathway for Decision Making

It has not previously been clear how the two distinct decision-making mechanisms associated with vmPFC and dACC might interact. The present study suggests that PCC is part of a final common pathway to action selection used by both systems.

The PCC region probably included areas 31 and 23, but it extended to the main branch of the cingulate sulcus at the point of its inflection into its marginal ramus (Figure 4B), where the caudal cingulate motor area is situated (Amiez and Petrides, 2012; Beckmann et al., 2009). Activity in this region, or just caudally, has been reported to resemble both that in vmPFC (Boorman et al., 2013; FitzGerald et al., 2009; Kolling et al., 2012) and that in dACC (Pearson et al., 2011). In our study, it was more closely coupled with vmPFC when risk bonus was low and the safer choice was taken, but it was more closely coupled with dACC when the riskier choice was taken and when the relative value of the riskier choice (V_riskier_ − V_safer_) increased (Figure 8). In other words, the region that PCC couples with during a decision is related to the signal it carries and the choice that subjects ultimately make. This means that, while there may be two parallel decision-making circuits dependent on dACC and vmPFC, both circuits have a serial element that converges in, or just posterior to, the caudal cingulate motor area in PCC.

Crucially, the competition between the two mechanisms associated with vmPFC and dACC was modulated by a third frontal region, the IFG (Figures 8 and S8). The IFG has often been identified with executive control (Swann et al., 2009), and the current results suggest that one way in which it might exert control is to regulate the relative activity in two parallel systems for decision making and the manner in which they interact with PCC. How exactly IFG is involved in the evaluative process itself is still unclear. Our results suggest that, if it has a causal role in promoting nondefault riskier choices, then its disruption would lead to taking safer, default choices. In agreement with the possibility that IFG or an adjacent lateral frontal region is involved in dynamic, context-dependent changes in decision making, one recent study applied transcranial magnetic stimulation in this vicinity and found that subjects were more likely to make socially unbiased decisions and to integrate considerations of reward magnitudes in the standard manner (Baumgartner et al., 2011), rather than taking the social context into consideration.

## Experimental Procedures

### Subjects

Eighteen subjects (nine women and nine men), aged 22–36 years, completed the task. They were paid £10 plus a performance-dependent bonus of between £15 and £30. Ethical approval was given by the Oxfordshire National Health Service Research Ethics Committee (local ethics code: 07/Q1603/11).

### Training

Before fMRI scanning, every subject was instructed in the task and played a shorter version of the task used in the fMRI experiment for about 10 min.

### Experimental Task

The behavioral task in the scanner consisted of 24 blocks, each composed of eight trials (192 decisions in total) in which the subjects had to decide between a safer option with a higher reward probability but a lower reward magnitude and a riskier option with a potentially higher reward magnitude but lower reward probability. There were eight decisions, and they were each presented once in each block in a randomized order that varied. In this way, we were conclusively able to show that dynamic changes in decisions occur, because of sensitivity to risk pressure, even when the exact same options were presented. Risk pressure varied because all eight decisions were associated with different values and were presented in different orders, with different outcomes, and in the context of different block target values (which the subjects had to reach in order to keep the points they won during the block).

Four target levels were used in the experiments. The different target levels helped ensure that risk pressure (see Introduction and the following section) had some parametric range. To equalize expected gains at the beginning of a block regardless of target level and to keep motivation relatively stable, we introduced a “multiplier,” which was displayed on top of the “target” line. The multiplier indicated a factor that would be used to multiply the points subjects won before they were added to the subject’s account if they reached the target. We chose the multiplication factor by applying our model (discussed in the next section) to generate equal expected gains at the first trial of a block. Simply put, if a participant had a high target to reach, all his points were multiplied (e.g., by 2) if he managed to reach it. Therefore, the subject should be equally motivated to perform the task when the targets were high, because the average payouts were similar. The use of the “multiplier” procedure ensured that the risk pressure signal that we observed (Figure 4) could not be explained away as a consequence of differing average reward expectations associated with different target levels. Further information about the experimental task and fMRI scanning is provided in the Supplemental Experimental Procedures.

### Behavioral Analysis

As explained in the Introduction, the target manipulation and block structure allowed us to compute three contextual variables. Trial number indexed how far through the block the subject had progressed. The second variable, risk pressure, indexed how many points a subject needed to gain on average on each remaining trial in order to reach the target. Risk pressure thus took into consideration the subject’s resources (the points they had earned prior to any given decision), the target number of points that had to be acquired in order to keep any earnings from the block, and the number of remaining foraging opportunities minus the number of trials that remained in the block.

(1) Riskpressure=(targetpoints−pointsalreadyearned)/trialsremaininginblock

In order to understand how risk pressure exerted an influence of decision making, it is first necessary to consider the relative value of riskier and safer options in the absence of any contextual modification. V_riskier_ − V_safer_, the value difference favoring riskier as opposed to safer choices, was calculated as follows:

(2i) $$V_{\text{riskier}}=\text{normalized}\left({\text{magnitude}}_{\text{riskier}}\right)+\text{normalized}\left({\text{probability}}_{\text{riskier}}\right)$$

(2ii) $${\text{V}}_{\text{safer}}=\text{normalized}\left({\text{magnitude}}_{\text{safer}}\right)+\text{normalized}\left({\text{probability}}_{\text{safer}}\right).$$

This is because we noticed that subjects acted as if they approximated V_riskier_ and V_safer_ by linearly combining each option’s component magnitude and probability rather than multiplying them as would be optimal (Figure S1). Nevertheless, there was a correlation (r > 0.86) between the value regressors we used and those we would have used had value been estimated multiplicatively. Note that both parameters (magnitudes and probability) were, separately, normalized by subtracting each mean and dividing by each SD. Finally, it is important to note that, while we follow convention in referring to these terms as “values,” it is, of course, the case that these values are inferred from subjects’ choices. Therefore, they are likely to be predictive of choices, but the question we investigate here is whether they are sufficient, in isolation, to explain choices or whether other contextual factors also influence decisions.

We, therefore, built a model examining the process of value modification due to contextual factors such as risk pressure. At its heart is the idea that, in the absence of risk pressure, it is optimal to combine information about both the probability and magnitude of a reward outcome associated with a choice but that, with increasing risk pressure, decision making should be guided increasingly by just the potential reward magnitudes at stake. Although we are not wedded to the precise parametrization of the model, the general aim of the approach is to find a principled and quantified way of modifying the decision rule, going from the unmodified decision rule that combines both reward probability and magnitude to a rule based exclusively on magnitudes. In the model, we use a parameter—the risk bonus scale—with scores ranging from 0 to 1 as the decision rule is changed from the unmodified version to the increasingly contextually modified version. We think that such adjustments of a decision rule provide an intuitive way to think about how an agent adjusts their behavior in a new situation. The contextual parameter risk bonus scale therefore captured the insight that participants should opt for the riskier choice, even if its associated reward probability was low, if it was going to be difficult for them to reach the block’s target level in the absence of that reward. At a risk bonus scale score of 0, there is no modification of the option values shown in Equation 2. At a risk bonus scale score of 1, the options’ values corresponded solely to their magnitudes.

The changes in the options’ values were formalized by adding an option bonus to each option’s raw value. This allowed estimation of a simple quantity that corresponded to how much an option’s value increased for a given level of risk pressure. The size of the option bonus depended on both (1) the risk pressure on a given trial but also on (2) the specific raw value of the option. The dependence on the specific raw value that each option possesses follows from the fact that high reward magnitude options, even when associated with low probabilities, have greater utility for reaching the target at the end of the decision sequence. The option bonus for a specific option A is calculated as:

(3) $$\text{option}\;{\text{bonus}}_{\text{A}}=\text{risk}\;\text{bonus}\;\text{scale}\times \left({\text{magnitude}}_{\text{A}}-{\text{magnitude}}_{\text{A}}\times {\text{probability}}_{\text{A}}\right).$$

The term in parentheses on the right side of Equation 3 can be thought of as an option-specific component of the option bonus. It is the difference between the number of points that could potentially be gained from that option (its magnitude) and the average points expected from that option (magnitude × probability; note that the product of magnitude and probability corresponds to the average value of the options under this optimal model). We used the option bonus to calculate modified model values of the options:

(4) $$\text{modified}\;\text{model}\;{\text{value}}_{\text{A}}=\left(M_{A}\times P_{A}\right)+\text{option}\;{\text{bonus}}_{\text{A}},$$

where M_A_ and P_A_ correspond to the magnitude and probability, respectively, of reward associated with option A. Alternatively, the modified value option A can be written as follows:

$$\text{modified}\;\text{model}\;{\text{value}}_{\text{A}}=\left(M_{A}\times P_{A}\right)+\text{risk}\;\text{bonus}\;\text{scale}\;*\left(M_{A}-\left(M_{A}\times P_{A}\right)\right)$$

or, again, alternatively as:

$$\text{modified}\;\text{model}\;{\text{value}}_{\text{A}}=\left(M_{A}\times P_{A}\right)+\left(\text{risk}\;\text{bonus}\;\text{scale}\;\times \left(M_{A}\times \left(1-P_{A}\right)\right)\right),$$

and decisions should be made as follows:

if (modified model value_A_ > modified model value_B_), then choose A;
else if (modified model value < modified model value_B_), then choose B.

So far, we have explained how the risk bonus scale was used in conjunction with the option’s reward probability and magnitude to estimate an option bonus for each option. It is necessary now to explain how the optimal risk bonus scaling itself was calculated. We simulated, for every trial, all unique decision sequences, each associated with a different risk bonus scale by calculating their modified values and using the aforementioned decision rule (Figure S3). For every unique decision sequence, generated with our value modification model, we could compute an end of block expected value. We defined the optimal risk bonus scaling as the risk bonus scale, which led to the decision sequence with the highest end of block value. It is important to note that, when doing so, we took into account that all net outcomes that fell short of the target value had a value of 0. Although we do not assume that participants were able to track the exact optimal risk bonus scaling, it served as an approximation of how the values of specific choices should be modified as a result of the context on a given trial. Task parameters were chosen to maximize its parametric range.

It is, furthermore, possible to calculate the risk bonus scale that leads to the point of equivalence for a given pair of options. In other words, at an optimal risk bonus scaling equal or above this value for an option pair, the riskier option should be preferred:

(5) $$\begin{array}{c} \text{equivalence}\;\text{risk}\;\text{bonus\_scale}=\left(M_{S}\times P_{S}-M_{R}\times P_{R}\right)/\left(M_{R}\times \left(1-P_{R}\right)-M_{S}\times \left(1-P_{S}\right)\right)\\ \text{or}\\ \text{equivalence}\;\text{risk}\;\text{bonus\_scale}=\left(M_{S}\times P_{S}-M_{R}\times P_{R}\right)/\left(\left(M_{R}-M_{S}\right)-\left(M_{R}\times P_{R}-M_{S}\times P_{S}\right)\right) \end{array},$$

where M_R_, M_S_, P_R_, and P_S_ refer to the reward magnitudes associated with the riskier and safer options and reward probabilities associated with the riskier and safer options, respectively.

By computing this value for all remaining decisions and rank-ordering decisions from the least to the most risky, we could estimate the value of all unique decision sequences and select the one that led to the highest end of block value. In all neural and behavioral analyses, the risk bonus scale used is, therefore, equal to the optimal risk bonus scaling in a given trial, i.e., the risk bonus scale that generates a sequence of future decisions that would lead to the highest expected value at the end of the block, taking into account the current context (risk pressure) and future prospects (set of options left and the pair presented).

The optimal risk bonus scaling is, therefore, a contextual parameter reflecting the degree of bias toward riskier choices that is optimal for a given context and applies to both options in a trial in the same way. The option bonus becomes larger for riskier choices, compared to safer choices, as the optimal risk bonus scaling increases, reflecting the riskier choices’ increased utility for reaching the target. Therefore, the option bonus can be understood as a combination of risk pressure to take a riskier choice and features of the specific option at hand.

Finally, we used one more parameter that we refer to as risk bonus (as distinct from optimal risk bonus scaling), which was used in neural and behavioral analyses. This was the difference in value modification in favor of the riskier choice compared to the safer choice. It was calculated using the optimal risk bonus scaling as:

(6) $$\text{Risk}\;\text{bonus}=\text{option}\;{\text{bonus}}_{\text{riskier}}-\text{option}\;{\text{bonus}}_{\text{safer}}.$$

Therefore, risk bonus reflects the relative change in value of the riskier choice, compared to the safer choice, which occurs as a function of risk pressure and the magnitude and probability characteristics of both choices in a given trial. We note that, in this regard, our model is an optimal model that serves to motivate definitions of terms but that real subjects may not be completely optimal. For example, if, instead, option bonuses were only adjusted as a function of their reward magnitudes (rather than as a function of both reward magnitudes and probabilities; Equation 3) then the resulting risk bonus regressor would be correlated at r = 0.96 with the regressor that we used.

In summary, the approach allows us to (1) examine decision making in the context of the varying impact of risk pressure and (2) conceive of the impact of risk pressure as a quantifiable modifying influence on a default decision-making process. However, we explore an alternative approach in the Supplemental Experimental Procedures that considers how an agent with sufficient experience of a set of contexts may use a reinforcement learning model to estimate the values of choices. A number of links between the approaches are identified and discussed.

## Supplemental Information

Document S1. Supplemental Experimental Procedures, Figures S1–S8, and Table S1
