# Supplementary material for: Multiple Neural Mechanisms of Decision Making and Their Competition under Changing Risk Pressure
Source: Neuron. 2014 Mar 5;81(5):1190–202. doi: 10.1016/j.neuron.2014.01.033 (PMC3988955; doi:10.1016/j.neuron.2014.01.033)
Supplement: Document S1. Supplemental Experimental Procedures, Figures S1–S8, and Table S1 [file mmc1.pdf]

Neuron, Volume *81*

Supplemental Information

Multiple Neural Mechanisms of Decision Making and Their Competition under Changing Risk Pressure

Nils Kolling, Marco Wittmann, and Matthew F.S. Rushworth

SUPPLEMENTAL DATA

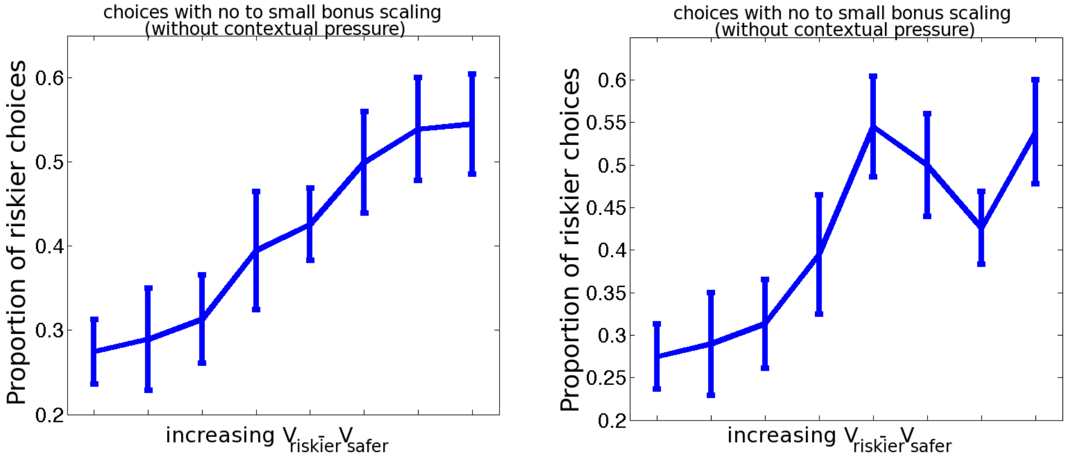

Figure S1 [related to Figure 1]: *Proportion of riskier choices taken as a function of relative value of the riskier choice ($V_{riskier}$-V_{safer}) in trials with little or no optimal risk bonus scaling (blue line in figure 1D). $V_{riskier}$ and V_{safer} were calculated as either the sum (left) or the product (right) of the options' reward magnitudes and probabilities.*

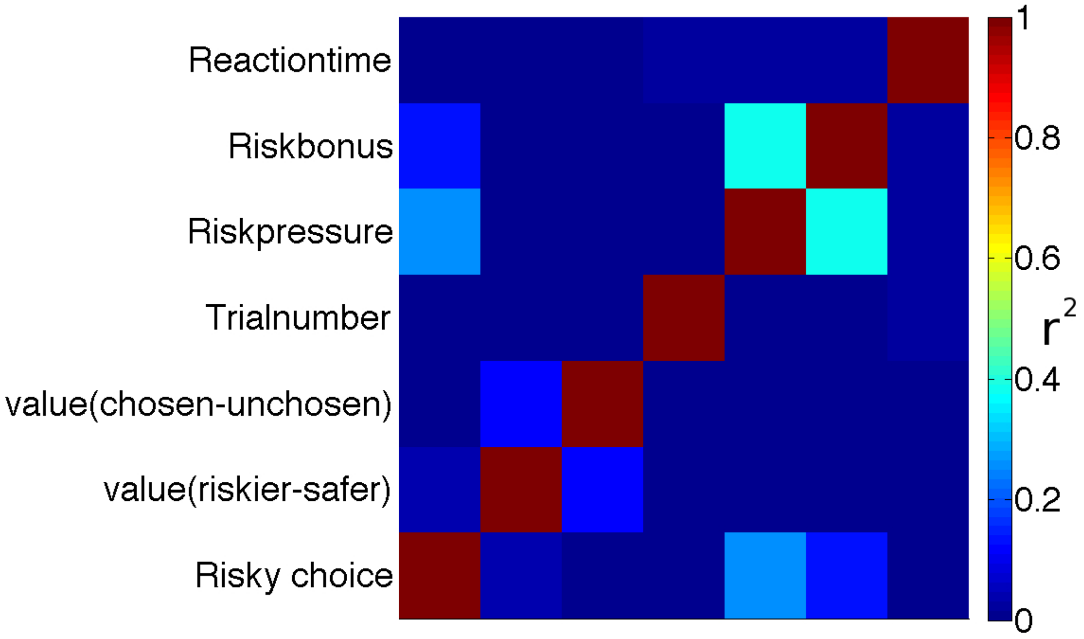

Figure S2 [related to Figure 1]: *overall correlations (r^2) between all regressors at the decision stage. The highest correlation is between the conceptually related factors of risk bonus and risk pressure. However, because risk bonus contains model based information about the optimal consequences of the risk pressure context on the evaluation of two specific choice options it is not confounded with the risk pressure itself.*

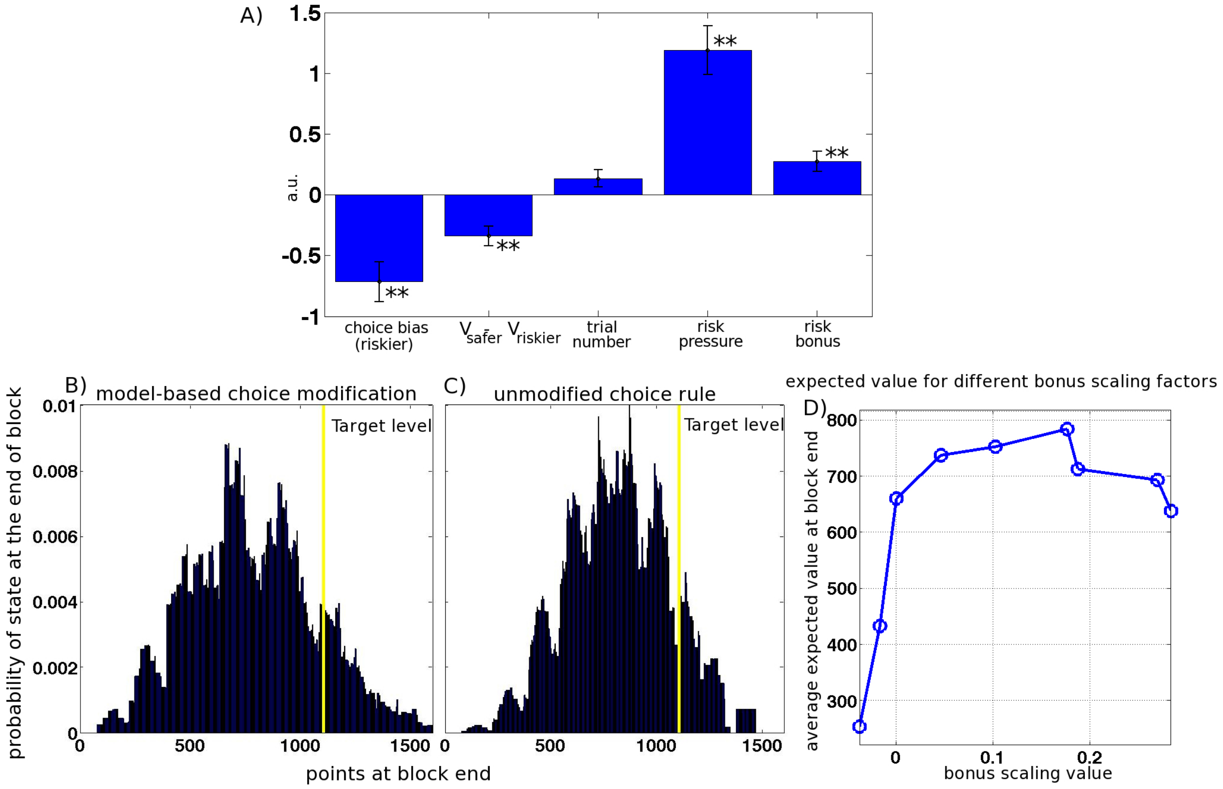

Figure S3 [related to Figure 2]: *A) Behavioral general linear model including both risk pressure and risk bonus. Although much of the behavioral variance is captured by including risk pressure as a regressor, risk bonus has a further and significant influence on behavior. (** p<0.01). Illustration of the results of decision sequences through the rest of a mini-block and calculation of optimal risk bonus scaling for an example trial. B and C) Histograms showing the probability distribution of block outcomes for two specific decision sequences (for illustrative purposes with a 20 point smoothing to represent uncertainty about precise final state). We considered only the part of the distribution that falls above the target level to calculate the expected value of a decision sequence, multiplied with a constant dependent on target level. The histogram in B) is based on the decision sequence with the maximal expected value for that trial, and therefore the one with the optimal risk bonus scaling, while the histogram in C) is based on the decision sequence suggested by the raw values of the choices. Note the difference in distribution ratio that falls to the right of the yellow target line. D) A plot showing the expected values of all unique decision sequences suggested by different risk bonus scaling values for a given trial. In this example a risk bonus scaling of around 0.18 is optimal.*

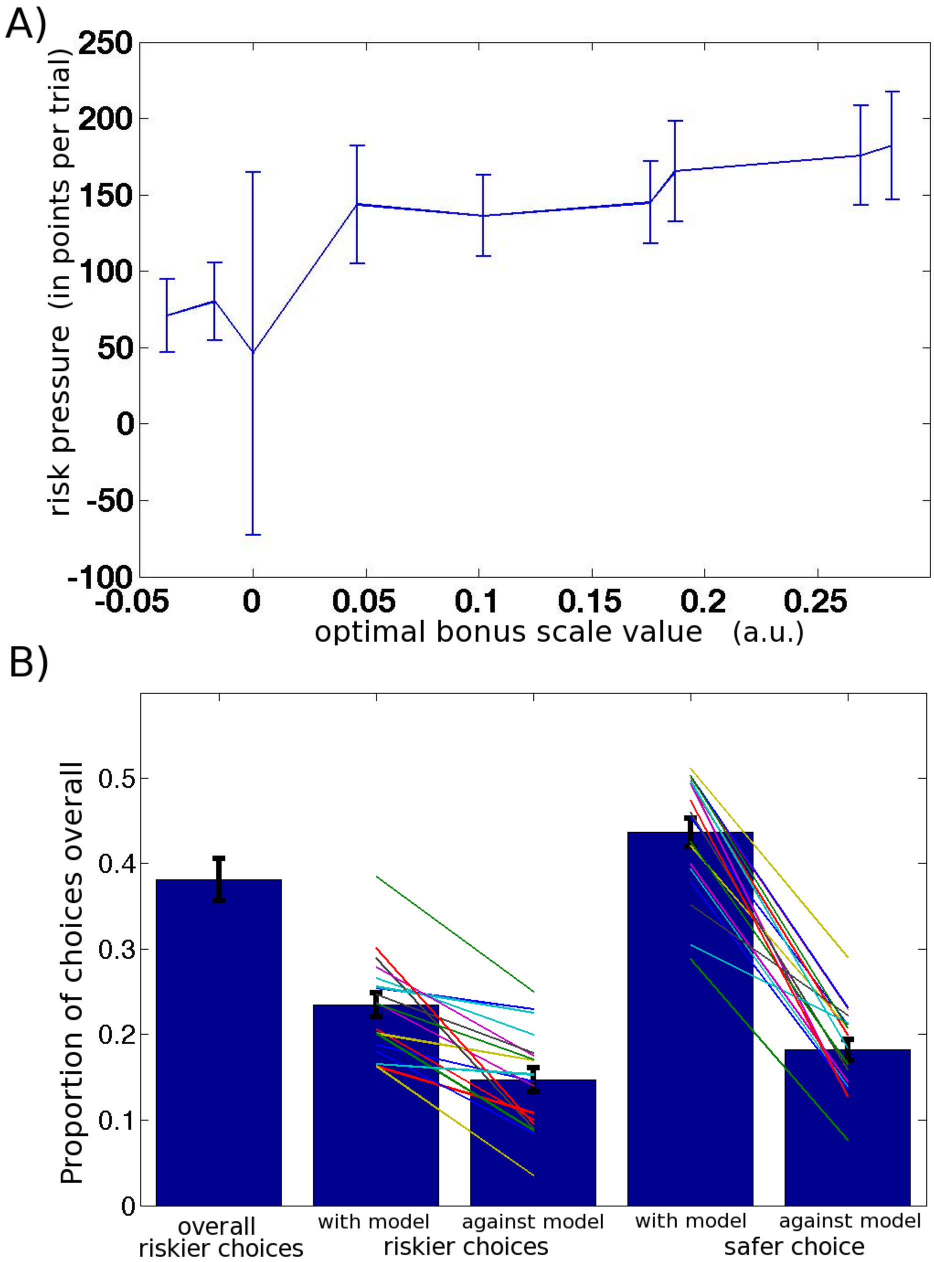

Figure S4 [related to Figure 2]: *A) The relationship between a trial's risk pressure and the optimal risk bonus scaling (as determined using the maximal expected value at the end of a block depicted in Figure S3). There is a clear relationship between both quantities although it is non-linear and not one-to-one in nature. A number of risk pressure levels entail no need to move towards a more risk-oriented decision strategy, whereas relatively small variations in high risk pressures should lead to quite large changes in the optimal risk bonus scaling and therefore predicted changes towards a more risk-oriented decision strategy. B) Overall proportion of riskier choices as well as a breakdown of decisions into four categories. Model conforming / opposing riskier and safer (lines indicate individual subjects).*

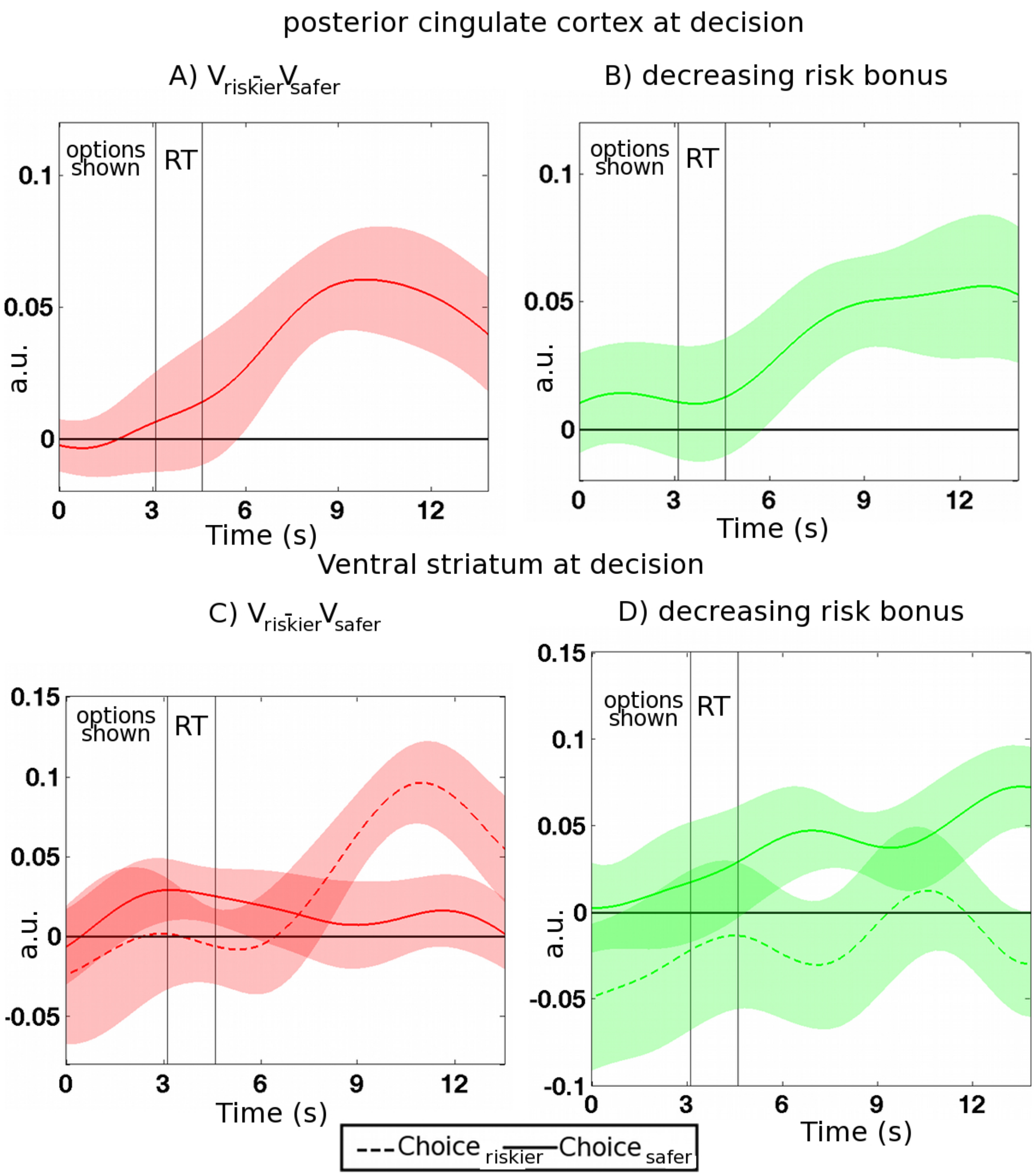

Figure S5 [related to Figure 4]: *Posterior cingulate cortex (PCC) for all decisions. A) Activity increased with the relative value of the riskier choice ($V_{riskier}$-V_{safer}) as in the dACC. B) However, PCC also activated when the risk bonus was reduced as did vmPFC. Time-course of the ventral striatum at decision split by choice (riskier or safer). C) The relative value of riskier choice ($V_{riskier}$-V_{safer}) only activated the ventral striatum when it was chosen. D) The reduction of the risk bonus activated the ventral striatum only when the safer choice was taken.*

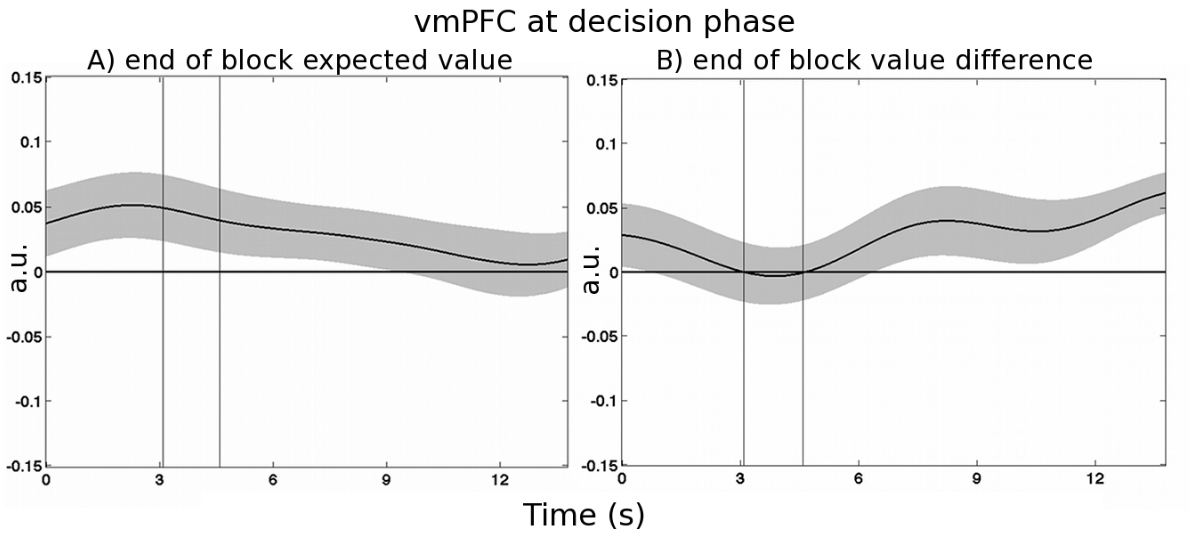

Figure S6 [related to Figure 5]: *In vmPFC there was no effect of the overall expected value at the end of the block as estimated by our model (left), nor was there any effect of the difference in the block's expected value as a function of the current choice being taken and the alternative choice (right).*

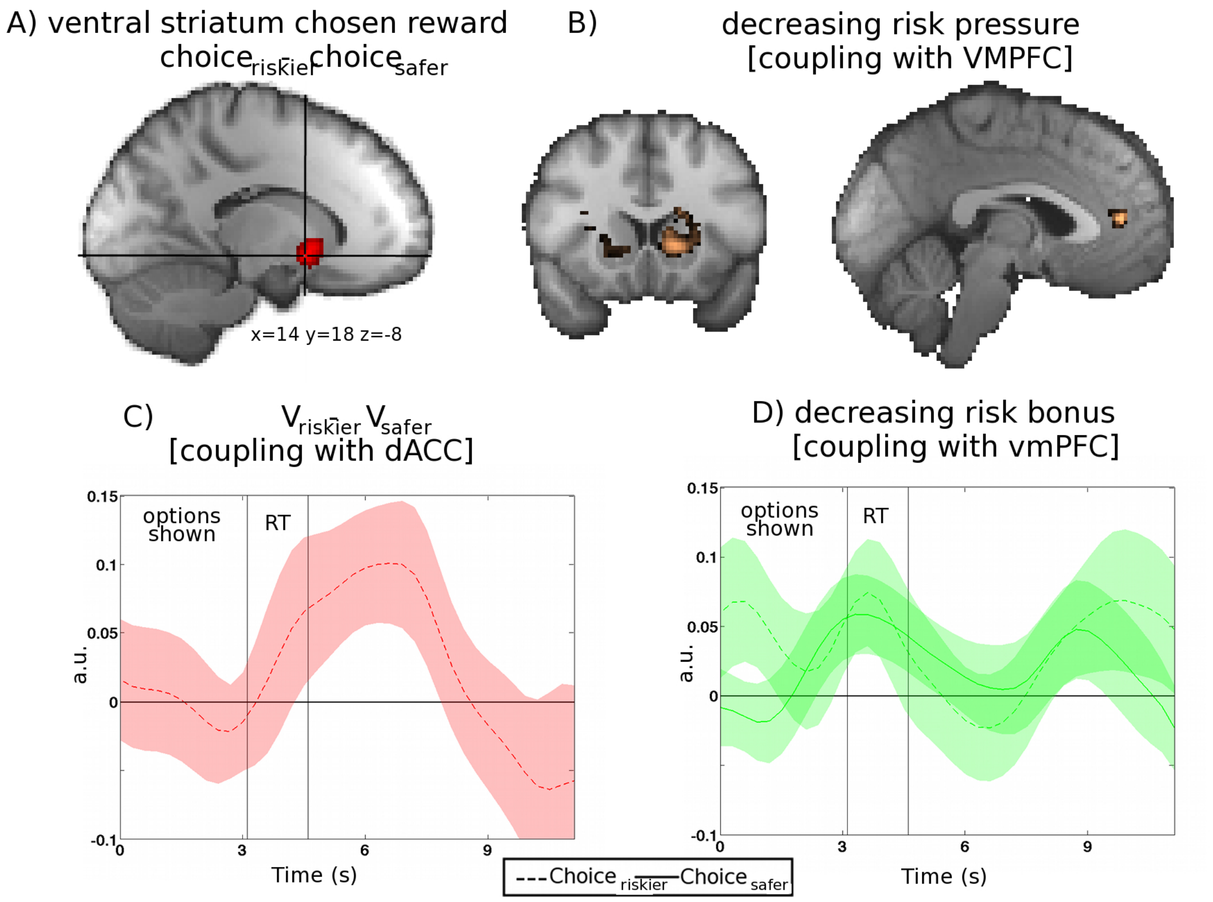

Figure S7 [related to Figure 8]: *A) The ventral striatum was more sensitive to rewards after taking the riskier choice than after taking the safer choice. It only encoded $V_{riskier}$-V_{safer} when the riskier choice was taken and decreasing risk bonus when the safer choice was taken). B) Physiological psychological interaction (PPI) analysis based on the time course of vmPFC activity from the ROI in figure 3A and inverse risk pressure [we used the inverse of the risk bonus regressor because vmPFC had been shown to be inversely related to the risk bonus in an earlier analysis (fig3A). Both, ventral striatum and pgACC (as opposed to dACC) exhibited higher functional connectivity with vmPFC when risk pressure*

was eased during the decision period. C) PPI between the ventral striatum and dACC (point of group peak activation related to $V_{riskier}$-V_{safer} (see figure 4). Note that the functional connectivity only changes as a function of $V_{riskier}$-V_{safer} when the riskier choice is taken. D) PPI between the ventral striatum and the vmPFC region from figure 3A. The vmPFC region was more active with decreasing risk bonus and it was also more coupled with the ventral striatum.

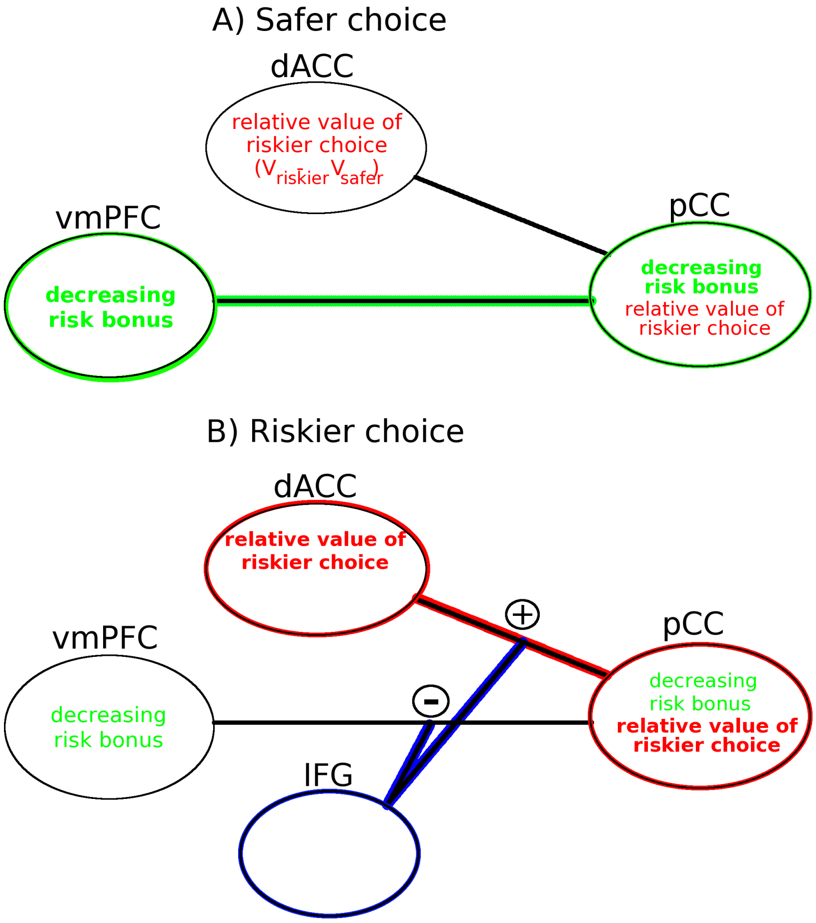

Figure S8 [related to Figure 8]*: A) The proposed network involved in generation of decisions in favor of the riskier choice. The three key brain areas, vmPFC, dACC, and PCC are indicated and the decision variable that was prominent in each area during the task is summarized. The brighter green font indicates that a negative risk bonus signal (mostly a negative effect on trials with a positive risk bonus) was present in vmPFC and this was associated with increased coupling with PCC when the safer choice was taken; in other words, as the risk bonus decreased, coupling of vmPFC with PCC increased when the safer choice was made. The paler red writing indicates that the signal representing the relative value of the riskier choice ($V_{riskier}$-V_{safer}) in dACC was not coupled with the PCC in the same decisions. B) The network for the generation of riskier choices. Now the brighter red font indicates that the PCC is more coupled with dACC, compared to vmPFC, as a function of $V_{riskier}$-V_{safer} and left IFG activity.*

Contrast	Z-coordinates MNI (mm)	Label	
$V_{riskier}$-V_{safer}	12,34,54	Right DLPFC	
	54,-60,38	Right PPC	
	0,-34,40	PCC	
	-50 -64 38	Left PPC	
	-2,28,36	dACC	
$Choice_{riskier}$-$Choice_{safer}$	-2,34,36	dACC	
	52,24,4	Right IFG	
	-28,24,-8	Left anterior insula	
	-48,30,-8	Left IFG	

Table S1 [related to Figure 4]: *List of peak activations for the corrected significant clusters for the whole brain contrasts.*

SUPPLEMENTAL EXPERIMENTAL PROCEDURES

To induce variability in choices, we used four different target levels (595, 930, 1035, 1105 points) and a block length of eight trials [the pairs of options associated with each decision each had a reward magnitude in points, and a reward probability (indicated as percentage): 100p x 90% vs 265p x 35%; 180p x 60% vs 260p x 35%; 145p x 75% vs 245p x 35%; 145p x 55% vs 350p x 20%; 115p x 90% vs 240p x 45%; 150p x 60% vs 190p x 45%; 170p x 75% vs 245p x 40%; 120p x 80% vs 210p x 30%]. With increasing target level the overall chances of exceeding the required points in a block decreased. In parallel, the optimal number of riskier choices, as predicted by our model, increased. The target levels used led to a good spread in the number of optimal riskier decisions (1-4 riskier decisions) predicted at the beginning of a block and thus to a good spread of the model parameter "optimal *risk bonus scaling*".

To ensure that the overall expected value of all blocks was the same even if the target levels were different, we introduced a multiplier that scaled the points earned when participants were successful and reached or exceeded the target at the end of the block. The multipliers for the four target levels were: target level of 595 points – 1.1 multiplier; target level of 930 points – 2.3 multiplier; target level of 1035 points – 3.3 multiplier, 1105 points – 4.2 multiplier. We presented participants with the same eight decisions in all blocks. Hence, we reduced variability between blocks that was due to features of the choices per se. Additionally we decorrelated the decision parameters (reward probability, reward magnitude, value of option, difference between values) as much as possible to be able to use them as separate regressors simultaneously in the fMRI analysis. Moreover, the Pascalian values (probability x magnitude) of six of the eight decisions favored the safer choice. This meant that because variation in the target parameter drove decisions towards the riskier choice, participants' behavior would exhibit an approximate balance between both safer and riskier choice decisions, which ensured sufficient sample sizes of both decision types for fMRI analysis.

The fMRI scan took approximately 55 minutes and was followed by a high-resolution structural scan and a field map acquisition for distortion adjustments. After scanning every subject completed a brief subjective rating questionnaire on the 'riskiness' of the eight decisions that had been presented in the fMRI task.

Trial time-course: Each trial started with the presentation of the safer and riskier choice options. After 3-4 seconds (Poisson distributed), a response cue (yellow question mark) indicated participants should make their decision (Figure 1A). The response cue stayed on until participants made a response using a button box. Reaction time (RT) was registered, defined as the time period between onset of the response cue and button press. We refer to the time period from the presentation of the decision to when participants made their choice as the decision phase. After a variable jitter of 3-7 seconds (Poisson distributed), in which the chosen option was highlighted on

the screen, feedback on both options was presented for 1-3 seconds (Poisson distributed). If the participant's choice was rewarded, then the points earned were graphically "added" to the "current points" bar (Figure 1A) indicating the amount of points gained so far in the block. We refer to this last time period in a trial as the feedback/outcome period.

An average trial took about 12 seconds and the inter-trial interval was jittered between 3 and 7 seconds (Poisson distributed). Within a block, each trial followed the previous one until the last trial of the block was reached. Afterward, a block outcome screen appeared for 4-7 seconds (Poisson distributed) that either displayed the gains of the participant in the block (if the target had been reached) or showed no gain for the block when the target was not reached.

Image data acquisition: Structural and functional MRI (fMRI) measurements were taken using a Siemens 3 Tesla MRI scanner. For the fMRI, we used a Deichmann echo-planar imaging (EPI) sequence (Deichmann et al., 2003) (time to repeat (TR): 3000 ms; 3**x**3**x**3mm voxel size; echo time (TE): 30ms; flip angle: 87°; slice angle of 15° with local z-shimming) to minimize signal distortions in orbitofrontal brain areas. This entailed orienting the window at 30° with respect to the AC-PC line.

Additionally for each participant, anatomical images were acquired with a T1- weighted MP-RAGE sequence, using a GRAPPA acceleration factor of 2 (TR: 2200 ms; TE: 4.53 ms; inversion time: 900ms; voxel size: 1**x**1**x**1 mm on a 176**x**192**x**192 grid) [same protocol as Kolling et. al., 2012].

Image data analysis: We used FMRIB's Software Library (FSL) (Smith et al., 2004) for image pre-processing and analysis. Functional images acquired were first spatially smoothed (Gaussian kernel with 5mm full-width half-maximum) and temporally high-pass filtered (3 dB cut-off of 100s). Afterward, the functional data were manually denoised using probabilistic independent component analysis (Beckmann and Smith, 2004), identifying and regressing out obvious noise components (Kelly et al., 2010). We used the Brain Extraction Tool (BET) from FSL (Smith, 2002) on the high-resolution structural MRI images to separate brain matter from non-brain matter. The resulting images guided registration of functional images in Montreal Neurological Institute (MNI)-space using affine registrations (7 degrees of freedom). The data was pre-whitened before analysis to account for temporal autocorrelations(Woolrich et al., 2001). Statistical analysis was performed at two levels. At the first level, we used an event-related general linear model (GLM) approach for each participant. On the second level, we used FMRIB's Local Analysis of Mixed Effects (FLAME) (Beckmann et al., 2003) with outlier de-weighting and tested the single group average. The main effect images are all cluster-corrected results with the standard threshold of z=2.3 and corrected significance levels of p=0.05.

FMRI analysis: We acquired up to 1200 volumes with 45 slices per subject. Constant regressors, modelled as boxcar functions, captured the three critical time phases that occurred in each trial: the decision phase, the outcome phase and the time period after every eighth trial in which the block outcome was presented. On each trial the time period indexed by the decision regressor began when the two options appeared on the screen and the duration lasted until the subject pressed a response button. Similarly, onset and duration of trial feedback and block outcome regressors covered the exact time the respective information was displayed on screen. We ran three separate GLM's. From the first:

$$\beta_0 + \beta_1(V_{riskier}\text{-}V_{safer}) + \beta_2(V_{chosen}\text{-}V_{unchosen}) + \beta_3(V_{riskier}+V_{safer}) + \beta_4(\text{risk bonus}) + \beta_5(\text{trial number}) + \beta_6[\log(\text{reaction time})]$$

we derived the $V_{riskier}$-V_{safer} signal shown in Fig.4B. The second GLM

$$\beta_0 + \beta_1(M_{riskier}\text{-}M_{safer}) + \beta_2(P_{riskier}\text{-}P_{safer}) + \beta_3(\text{risk pressure}) + \beta_4(\text{trial number}) + \beta_5[\log(\text{reaction time})]$$

was very similar but now $V_{riskier}$-V_{safer} effects were split by choice (riskier or safer) and probability ($P_{riskier}$-P_{safer}) and magnitude ($M_{riskier}$-M_{safer}) differences were modeled separately . Although modeling riskier and safer choices separately revealed interesting results that are reported

(Figs.4C, 5A), the independent modeling of probability and magnitude did not lead to new insights. In a third similar GLM we focused on splitting the trials with active risk bonus by choice (riskier or safe) for the value and risk bonus regressors. For each trial type (safer option chosen or riskier option chosen) we therefore had the following GLM:

$$\beta_0 + \beta_1(V_{riskier}) + \beta_2(V_{safer}) + \beta_3(\text{risk bonus}) + \beta_4(\text{trial number}) + \beta_5[\log(\text{reaction time})]$$

This revealed the risk bonus effect in vmPFC (Fig.3A) and effect of the number of remaining trials (Fig.3B). In all analyses we excluded all decisions taken after the target was reached as well as decisions when the target was beyond reach.

All dACC activity time-courses were taken from a single dACC ROI (MNI coordinates x=-2, y=28, z=36) where the group level effects of risk pressure and $V_{riskier}$-V_{safer} overlapped (also peak of overall $V_{riskier}$-V_{safer} effect). PCC, vmPFC, and IFG ROIs were centered at the group peak contrast effect that first identified them. The GLMs used in constructing activity time courses during decision periods included similar regressors to those used in the whole brain analyses: $V_{riskier}$-V_{safer}, trial number, log(reaction time), risk pressure and risk bonus.

We also conducted PPI analyses (Friston et al., 1997) to investigate functional connectivity between vmPFC and PCC and between dACC and PCC during the decision period. For the first of these analyses we generated a demeaned BOLD time-course regressor from the vmPFC as well as an interaction term with the demeaned psychological regressor [we used the inverse of the risk bonus regressor because vmPFC had been shown to be inversely related to the risk bonus in an earlier analysis (Fig3a)] separated according to choice (riskier or safe), to generate two PPI interaction terms corresponding to the trials in which riskier and safer choices were made. The main effects of the same two psychological regressors were also included in the GLM. In addition we also included the relative value regressor $V_{riskier}$-V_{safer} split by choice and log(reaction time). The PPI analysis for the dACC PPI analysis was conducted in an analogous manner but using a physiological regressor derived from the dACC ROI and a psychological regressor of $V_{riskier}$-V_{safer}. For the PPI time-courses, we subtracted the global mean (the average from all voxel at a specific time point) from the individual time-course of each region investigated, to avoid positive results due to global correlations.

To investigate interactions between dACC-PCC and vmPFC-PCC as a function of IFG activity we took the difference between the dACC-PCC PPI regressor and the vmPFC-PCC PPI regressor and multiplied it with the normalized, time-course from the left IFG, seeded at the group peak effect of the $choice_{riskier}$ – $choice_{safer}$ contrast (Fig.4A).

General linear models of fMRI analysis in ROIs:

The time-courses were derived from regions-of-interest (ROI), a sphere of 3 voxel radius, identified in Montreal Neurological Institute (MNI) standard space on the basis of the whole group analysis, calculating a mean time-course within a ROI in each subject individually and the coordinates were then transformed to individual subject space by using the same linear registration as in the initial analyses. We then oversampled the time-course by ten and created epochs from the beginning of an event onward and applied a GLM to every pseudo-sampled time point separately. By averaging the resulting β weights across subjects we created the time-courses shown (the standard errors were calculated between subjects).

Near-Optimal Model and Supplemental behavioral analysis:

A GLM with both risk pressure and risk bonus reveals that both regressors explain unique behavioral variance (Figure S3A). The value difference term (V_{safer}-$V_{riskier}$) is the fixed difference in preference between the safer and riskier option and *trial number* refers to the current trial's position in a block. The *risk pressure* term summarizes how the environmental context should lead to riskier choices. The *risk bonus* term, however, encapsulates a model-based interaction of both the contextual factor of risk pressure and features of the specific choice options available on the current trial. It predicts how participants should take risks that are dependent on how much the risk pressure on a trial should lead to a difference in evaluation of the specific current options also

taking into account future possibilities for taking such risks. It can be seen that it explains additional variance in behavior over and above that explained by risk pressure. Another illustration of this can be seen in figure 1 E.

Figure S3B-D depicts how the *optimal risk bonus scaling* was determined based on the decision sequences for an example trial. Figure S4A depicts the relationship between the *optimal risk bonus scaling* and *risk pressure*. Although the *optimal risk bonus scaling* has a strong relationship with the *risk pressure*, the relationship is non-linear; variations in *risk pressure* do not always lead to a change *in optimal risk bonus scaling*, for example several levels of *risk pressure* are associated with an *optimal risk bonus scaling* of zero indicating that there should be no contextual modification of the options' values. Small changes in *risk pressure*, when *risk pressure* is already high, lead to large increases in *optimal risk bonus scaling*.

The model is very nearly optimal. The decisions taken by our model after taking into account risk pressure and by a truly optimal model differ in less than 7% of cases. More importantly the divergences in expected value at the end of the block that would follow from taking a sequence of actions according to our risk sensitive model or according to a truly optimal model are very small; the expected values of the choices our model generates, after taking into account risk pressure, are highly correlated (r>0.98) with the expected values of the choices generated by a truly optimal model (in which all possible decision sequences are computed and compared according to their expected values). The reason we use our model and do not simply take the completely optimal decision sequence, is that our model offers a simple account of how context might lead to a simple modification of a choice's value that might in turn lead to a change in the decision that is taken.

The reason a small number of minor divergences occur between our model and the completely optimal decision sequence has to do with the hard target constraint; in some cases very specific outcome combinations add up to generate a slightly higher overall expected value than our model. This is because our model is constrained to envisage that the level of risk-taking in the rest of the block will be consistent with the level of risk-taking in the current trial when it computes the level of risk-taking that is appropriate in the current trial. Thus it computes a uniform level of risk-taking for the entire sequence of decisions that remain before the block end. By contrast an optimal model is not constrained by any concept of risk taking and simply finds the decision sequence that gives the highest expected value. In this way a small number of specific decision sequences become very slightly more optimal because they lead to an expected value that just exceeds the target. However, because the gain in expected value is rather minimal and can most likely only be computed with a very precise knowledge of all specific magnitudes and probability in the decision sequence we thought that it was unlikely that participants could make use of this information. Moreover we found no empirical evidence that they could do so. Indeed, on average, for the 7% of decisions in which the optimal sequence and our model diverged from each other, about half were made in accordance with our model and half in accordance with the optimal sequence.

We compared participants' behavior to the predictions of the model (Figure S4B). Participants were more likely to make model-conforming safer choices than they were to make model-conforming riskier choices. However, riskier choices were still more likely to conform to the model prediction than not. This shows that even though participants were not completely optimal, they integrated over value and contextual factors in a way predicted by our model, with a slight overall bias against the riskier option.

Reinforcement learning approach

An alternative approach to our task is a reinforcement learning one. Such an analysis has the appeal that it aligns naturally with other past studies using state-based reinforcement learning to explain neural data and behavioral performance.

It would be feasible to use a reinforcement learning approach to analyze the behavior recorded in our paradigm. Such a model approach might represent all combinations of the current number of points acquired and of trial number and it would estimates expected overall block end values separately for each target level. However, when related reinforcement learning-based approaches have been used in the past they have typically not incorporated knowledge of which option pairs have actually been observed in past decisions; they therefore incorporated no expectations about how options might be paired in future decisions or knowledge of the specific

sequence of decisions in a block). In order to incorporate this information it would be necessary additionally to hold independent state space representations for every possible combination of sequences.

We therefore used a state space-based reinforcement learning model of this type. The starting point of the model is to estimate the values of all possible block end states taking into account the four different target levels, i.e. making all below target values zero. We subsequently computed the value of all earlier states in blocks, one trial back at a time, giving equal likelihoods of all option pairing appearing in any decision, by looking up the values of the later states that would follow on from them as a function of the state space map. Furthermore, we made the assumption that in every decision the option that would lead to the higher next state would be selected. Doing so from the last to the first trial, it is possible to give the current state a value defined by the state (trial number and point count) as estimated from the model and also find such a state estimate for each the two options if they are chosen in the decision. Since the future state is different for losses and wins, we need to compute two future state space values for each option. The two future state values are then averaged by using the probabilities of winning for each option i.e. by multiplying the value of each state with its probability, which is the expected future state value for both available options, The difference between both options' future state values is the state value difference and, according to such an approach, should be the decision variable. The model assumed equally likely redraws of all eight trial types for every future trial.

We then compared several key value estimates and decision variables using this state-based reinforcement learning model with those derived from our risk pressure-risk bonus model which is explained in Methods and above. In order to make the comparison fair we used a slightly modified version of our risk pressure-risk bonus model, which also assumed equally likely redraws of all eight trial types for each future decision (r=0.79 between future block end value differences). We estimated:

1) future state space value differences/future block end value differences. The future state space value for each of the two options on each decision under the reinforcement learning model and we then determined the difference between the future state space values. We then found the comparable term using our risk pressure-risk bonus based model by determining the expected value at the end of the block for each choice that might be taken in a decision and then determining the difference between the expected values for each pair of options offered in a decision. There was a high correlation between reinforcement learning-based future state space value differences and risk pressure-risk bonus model based future block value differences (r=0.95).

2) overall expected value at the end of the block. The overall expected value at the end of the block estimated under the reinforcement learning model and risk pressure-risk bonus model were highly correlated (r=0.97).

3) Q-action values and option values for specific choices. The Q-action values associated specific options under the reinforcement learning approach and the option values used by the risk pressure-risk bonus model were highly correlated (r=0.97).

Thus both approaches generate almost equivalent estimates of ultimate or long term value expectations at the block end and something like such estimates were found to be represented in the dACC (figure 5B). However, only the risk pressure-risk bonus approach also has parameters relating to risk pressure and the risk-based value modification of option values in a specific decision and such terms were useful for describing both behavior and the pattern of activity recorded in dACC, vmPFC,PCC, and IFG. Moreover, none of these areas appeared to carry a future state space value difference code. In other words we did not find an area that represented the difference between the expected value at the block end if one option or another were taken on the current trial. This could be due to the feedback given after every trial which might make the actual end of block states seem relatively unpredictable in comparison to the outcome for each component decision in the block and which might have led to a decision-making process that was more guided by current option values and a contextually based value modification. Nevertheless a reinforcement learning and state-based approach might provide additional important insights under different conditions, for example if subjects always progressed through the same fixed order of decisions.

Linear approximation of choice values
Participants appeared to combine probability and magnitude linearly. This is apparent when the proportion of riskier choices made as a function of the relative value of the riskier choice ($V_{riskier}$-V_{safer}) is plotted when values are computed as a linear approximation or as the true product of the component reward magnitudes (Figure S1) in trials with little or no optimal risk bonus scaling according to our model. Choices are not so well explained by choice values that computed by multiplication.

Supplemental fMRI results
If the decisions participants made were not just motivated by the values of the immediately available choices but also by values of the outcomes expected at the end of the block then activity encoding block end values is expected. We found such a signal in dACC (main manuscript, Fig.5B). However, in vmPFC there was no effect of the overall expected value at the end of the block as estimated by the model nor was there any effect of the difference in the block's expected value as a function of the current choice being taken and the alternative choice (Figure S6).

As shown in figure 4B the posterior cingulate cortex (PCC) carried a $V_{riskier}$-V_{safer} signal (time course shown in Figure S5A), but was also, like vmPFC, more active when the risk bonus decreased (Figure S5B). This is further evidence for its role in representing information coming from both vmPFC and dACC and possibly its connectivity with both brain regions is a way of mediating different decision strategies, i.e. ways of weighing up different properties of current decision alternatives.

Activity and coupling changes involving the ventral striatum also occurred during task performance (Figures S5C+D and S7). In summary, ventral striatum represented $V_{riskier}$-V_{safer} only when the riskier choice was taken. By contrast risk bonus had a negative impact on the striatum when safer choices was taken. The chosen reward activated the ventral striatum more when the riskier choice was taken (Figure S7A). Like the PCC, connectivity with dACC was modulated by $V_{riskier}$-V_{safer} only when the riskier choice was taken. However, the same was not true for the vmPFC connectivity. If anything, ventral striatal-vmPFC coupling decreased as the risk bonus increased although the coupling change, might have been more closely related to what is shown in Figure S7, i.e. more related to changes in risk pressure.

We also looked for other evidence that vmPFC coupling with other areas might increase and decrease as risk pressure waxed and waned (Figure S7B). We first looked at vmPFC functional connectivity as a function of decreased risk pressure. We predicted that if decreasing risk pressure increased the degree of vmPFC involvement in choice then its functional connectivity would increase correspondingly. Decreasing risk pressure indeed led to increased coupling between vmPFC and pregenual ACC (pgACC). Note that, while pgACC is in the cingulate cortex it is separate from dACC and appears to have a different function; activity in the pgACC has previously been observed in relation to the degree to which people are prepared to adopt strategies other than the simplest or default one and are prepared to search through the environment for better alternative strategies in the optimal manner (Kolling et al., 2012) (Figure S7B). The human pgACC may correspond to a pregenual ACC area in the macaque in which activity is related to the manner in which the costs and benefits of courses of action are compared (Amemori and Graybiel, 2012).

Supplemental References

Amemori, K., and Graybiel, A.M. (2012). Localized microstimulation of primate pregenual cingulate cortex induces negative decision-making. Nat Neurosci 15, 776-785.
Beckmann, C.F., Jenkinson, M., and Smith, S.M. (2003). General multilevel linear modeling for group analysis in FMRI. Neuroimage 20, 1052-1063.
Beckmann, C.F., and Smith, S.M. (2004). Probabilistic independent component analysis for functional magnetic resonance imaging. IEEE Trans Med Imaging 23, 137-152.
Deichmann, R., Gottfried, J.A., Hutton, C., and Turner, R. (2003). Optimized EPI for fMRI studies of

the orbitofrontal cortex. Neuroimage 19, 430-441.
Kelly, R.E., Jr., Alexopoulos, G.S., Wang, Z., Gunning, F.M., Murphy, C.F., Morimoto, S.S., Kanellopoulos, D., Jia, Z., Lim, K.O., and Hoptman, M.J. (2010). Visual inspection of independent components: defining a procedure for artifact removal from fMRI data. J Neurosci Methods 189, 233-245.
Kolling, N., Behrens, T.E., Mars, R.B., and Rushworth, M.F. (2012). Neural mechanisms of foraging. Science 336, 95-98.
Smith, S.M. (2002). Fast robust automated brain extraction. Hum Brain Mapp 17, 143-155.
Smith, S.M., Jenkinson, M., Woolrich, M.W., Beckmann, C.F., Behrens, T.E., Johansen-Berg, H., Bannister, P.R., De Luca, M., Drobnjak, I., Flitney, D.E., *et al.* (2004). Advances in functional and structural MR image analysis and implementation as FSL. Neuroimage 23 Suppl 1, S208-219.
Woolrich, M.W., Ripley, B.D., Brady, M., and Smith, S.M. (2001). Temporal autocorrelation in univariate linear modeling of FMRI data. Neuroimage 14, 1370-1386.
